# Enhanced Microbial Bile Acid Deconjugation and Impaired Ileal Uptake in Pregnancy Repress Intestinal Regulation of Bile Acid Synthesis

**DOI:** 10.1002/hep.30661

**Published:** 2019-05-21

**Authors:** Caroline Ovadia, Alvaro Perdones‐Montero, Konstantina Spagou, Ann Smith, Magali H. Sarafian, Maria Gomez‐Romero, Elena Bellafante, Louise C.D. Clarke, Fouzia Sadiq, Vanya Nikolova, Alice Mitchell, Peter H. Dixon, Natalie Santa‐Pinter, Annika Wahlström, Shadi Abu‐Hayyeh, Julian R.F. Walters, Hanns‐Ulrich Marschall, Elaine Holmes, Julian R. Marchesi, Catherine Williamson

**Affiliations:** ^1^ Division of Women and Children's Health King's College London London United Kingdom; ^2^ Section of Biomolecular Medicine, Division of Computational & Systems Medicine, Department of Surgery & Cancer, Faculty of Medicine Imperial College London London United Kingdom; ^3^ School of Biosciences Cardiff University Cardiff United Kingdom; ^4^ Division of Digestive Diseases Hammersmith Hospital, Imperial College London London United Kingdom; ^5^ Institute of Medicine, Department of Molecular and Clinical Medicine and Wallenberg Laboratory University of Gothenburg Gothenburg Sweden; ^6^ Centre for Digestive and Gut Health, Department of Surgery and Cancer Imperial College London London United Kingdom

## Abstract

Pregnancy is associated with progressive hypercholanemia, hypercholesterolemia, and hypertriglyceridemia, which can result in metabolic disease in susceptible women. Gut signals modify hepatic homeostatic pathways, linking intestinal content to metabolic activity. We sought to identify whether enteric endocrine signals contribute to raised serum bile acids observed in human and murine pregnancies, by measuring fibroblast growth factor (FGF) 19/15 protein and mRNA levels, and 7α‐hydroxy‐4‐cholesten‐3‐one. Terminal ileal farnesoid X receptor (FXR)‐mediated gene expression and apical sodium bile acid transporter (ASBT) protein concentration were measured by qPCR and western blotting. Shotgun whole‐genome sequencing and ultra‐performance liquid chromatography tandem mass spectrometry were used to determine the cecal microbiome and metabonome. Targeted and untargeted pathway analyses were performed to predict the systemic effects of the altered metagenome and metabolite profiles. Dietary CA supplementation was used to determine whether the observed alterations could be overcome by intestinal bile acids functioning as FXR agonists. Human and murine pregnancy were associated with reduced intestinal FXR signaling, with lower FGF19/15 and resultant increased hepatic bile acid synthesis. Terminal ileal ASBT protein was reduced in murine pregnancy. Cecal bile acid conjugation was reduced in pregnancy because of elevated bile salt hydrolase‐producing *Bacteroidetes*. CA supplementation induced intestinal FXR signaling, which was not abrogated by pregnancy, with strikingly similar changes to the microbiota and metabonome as identified in pregnancy. *Conclusion*: The altered intestinal microbiota of pregnancy enhance bile acid deconjugation, reducing ileal bile acid uptake and lowering FXR induction in enterocytes. This exacerbates the effects mediated by reduced bile acid uptake transporters in pregnancy. Thus, in pregnant women and mice, there is reduced FGF19/15‐mediated hepatic repression of hepatic bile acid synthesis, resulting in hypercholanemia.

**Abbreviations**
ASBT: apical sodium dependent bile acid transporter
BSH: bile salt hydrolase
C4: 7α‐hydroxy‐4‐cholesten‐3‐one
CA: cholic acid
CDCA: chenodeoxycholic acid
*Cyp7a1*: cytochrome P450 family 7 subfamily A member 1
DCA: deoxycholic acid
FGF: fibroblast growth factor
FXR: farnesoid X receptor
LCA: lithocholic acid
LPE: lysophosphatidylethanolamine
MCA: muricholic acid
OST: organic solute transporter
PM5S: epiallopregnanolone sulfate
RXR: retinoid X receptor
SHP: small heterodimer partner
TMCAs: tauromuricholic acids
UPLC‐MS/MS: ultra‐performance liquid chromatography tandem mass spectrometry

In normal human pregnancy, there is a change in metabolism with advancing gestation that results in elevated serum bile acids,^1^ hypercholesterolemia, and hypertriglyceridemia^2^ in the third trimester. These gestational alterations in lipid metabolism are essential to ensure nutrient provision for the growing fetus and are associated with altered expression of key genes in pathways that control bile acid and lipid homeostasis. Serum and hepatocyte bile acid concentrations are elevated on day 18 of murine pregnancy,^3^, ^4^ likely secondary to gestational increases in 17β‐estradiol^5^ and sulfated progesterone metabolites.^6^ These endocrine changes cause reduced function of hepatic farnesoid X receptor (FXR); the sulfated progesterone metabolite, epiallopregnanolone sulfate (PM5S), is a partial agonist of FXR,^7^ whereas 17β‐estradiol–bound estrogen receptor α directly interacts with FXR to repress downstream transcription.^5^ Bile acids also signal through enterocyte and enteroendocrine L‐cell receptors to influence bile acid homeostasis and the release of hormones that impact gestational lipid and bile acid metabolism. Activated FXR in enterocytes dimerizes with retinoid X receptor (RXR), causing induction of rodent ileal bile acid binding protein,^8^ organic solute transporters (OSTs; OSTα and OSTβ),^9^ secretion of the hormone fibroblast growth factor (FGF) 15 in mice, FGF19 in humans,^10^ and small heterodimer partner (SHP)‐mediated repression of apical sodium‐dependent bile acid transporter (ASBT).^11^ Bile‐acid–binding and activation of the G‐protein‐coupled receptor, Takeda G‐protein‐coupled receptor 5 (TGR5), expressed by enteroendocrine cells within the gut, results in release of glucagon‐like peptide 1 (GLP1).^12^ Thus, luminal bile acids have a profound effect on bile acid, lipid, and glucose homeostasis through interaction with different receptors in a range of tissues.^13^

The composition of the gut microbiota influences the luminal concentration and conjugation of specific bile acids and consequent expression of enzymes that control intestinal and hepatic bile acid transport and metabolism through FXR‐dependent pathways (reviewed in a previous work^14^). In brief, when compared to germ‐free mice, conventionally reared animals had higher serum and fecal bile acid concentrations, more deconjugated bile acids in the distal gut, and a lower proportion of taurine‐conjugated bile acids in the proximal and distal gut^15^ and other tissues, including the kidney and heart.^16^ The potency of bile acid species as FXR agonists differs,^17^ chenodeoxycholic acid (CDCA), cholic acid (CA) and their taurine conjugates are FXR agonists, whereas the taurine‐conjugated α and β muricholic acids (MCAs) are FXR antagonists.^15^ Furthermore, alterations in the luminal concentration of specific bile acids will affect the abundance of particular microbes within the gut lumen; for example, *Bilophila wadsworthia* grows preferentially in the presence of high bile acid concentrations,^18^ whereas deoxycholic acid (DCA) and CDCA are toxic to the growth of *Bifidobacterium breve*, *Blautia coccoides* JCM 1395^T^, and *Bacteroides thetaiotaomicron* DSM 2079^T^.^19^ FXR activity in the intestine has been shown to regulate bacterial growth: Administration of the synthetic FXR agonist, GW4064, after bile duct ligation (when bacterial overgrowth is common) reduced both aerobic and anaerobic bacteria in the ileum and cecum; this effect did not occur in *Fxr^–/–^* mice.^20^

Studies of pregnancy typically demonstrate alterations in the intestinal gut microbiota through gestation; two human studies reported marked alterations in the third trimester compared with the first,^21^, ^22^ with resultant metabolic changes when third trimester feces was used to colonize germ‐free mice.^21^ A longitudinal study in mice demonstrated that changes to the gut microbiota occurred early in gestation^23^; however, a longitudinal human study of 49 women did not show gestational alterations in gut bacterial composition.^24^

In this study, we sought to determine whether altered enterohepatic feedback contributes to the hypercholanemia of pregnancy, including whether gestational alterations in the human gut microbiota and intestinal metabonome could contribute. We then utilized a murine model of CA feeding, which has similar phenotypic differences in lipid composition and hepatic steatosis to pregnancy,^3^ to establish whether enterocyte exposure to an FXR ligand abrogated these gestational effects.

## Materials and Methods

### Human Samples

The study conformed to the 1975 Declaration of Helsinki guidelines; permission was obtained from the ethics committees of Hammersmith Hospitals NHS Trust, London (08/H0707/21 and 11/LO/0396) and Sahlgrenska University Hospital, Gothenburg (Dnr 536‐14). Written informed consent was received from participants before inclusion.

Fecal samples were obtained from 14 women with uncomplicated pregnancies and 9 nonpregnant healthy women. Women were restricted to those with spontaneously conceived third trimester singleton pregnancies, without pregnancy complications and who had not taken antibiotics for the duration of the pregnancy. Fecal samples were frozen at −80^o^C within 24 hours of collection.

Serum samples for analysis of FGF19 and 7α‐hydroxy‐4‐cholesten‐3‐one (C4) were taken from women given a standardized diet from 6:00 pm the preceding day, with venepuncture performed at 8:00 am (fasting) and 3:00 pm. Participants were given fixed meals at 6:00 pm, 8:00 am, and 12:00 pm and remained sedentary throughout. Of the participants, 14 were nonpregnant (with previously uncomplicated pregnancies) and 24 had uncomplicated pregnancies. Blood samples were obtained in Vacutainer Serum Separation Tubes and after 30 minutes were centrifuged at 1,700*g* for 10 minutes. Serum was aliquoted and stored at −80^o^C. Serum analyses for FGF19 and C4 were performed as described,^25^ using enzyme‐linked immunosorbent assay (ELISA; FGF19 Quantikine ELISA kit, Cat. No. DF1900; R&D Systems, Minneapolis, MN), and high‐performance liquid chromatography following solid‐phase extraction for C4.

### Animal Handling

Experiments were conducted according to the UK Animals (Scientific Procedures) Act of 1986 and with protocols approved by the King's College London Animal Studies Committee. Female C57BL/6 mice (Charles River, Wilmington, MA) were housed in the same room within clean facilities, with a 12‐hour light cycle. Cages housed 3 mice per cage, all from the same dietary group. Mice were fed a normal chow diet (RM3 control diet, Special Diets Services, Essex, UK) *ad libitum* until mating, at which time half the mice had a diet supplemented with 0.5% CA (LBS Biotechnology, Horley, UK) until delivery. Mice were sacrificed on day 0 or day 18 of pregnancy, resulting in n = 6‐8 per group. Separate cohorts of C57BL/6 mice were sacrificed at increasing gestational ages (days 2, 7, 10, 14, and 18; n = 6‐7 per time point), as described.^26^

At sacrifice, murine ceca, ileum, duodenum, gall bladder, and liver were harvested and snap frozen on dry ice and subsequently stored at −80^o^C. While frozen, ceca were longitudinally split and content dissected from overlying intestinal tissue.

### DNA Extraction

DNA was extracted from cecal content using Qiagen Tissuelyser II bead beating and a QiaAMP Fast DNA Stool Mini Kit (Qiagen, Venlo, Netherlands), according to the manufacturer's instructions.

### Metagenomics Analysis

The 29 murine samples of extracted DNA were treated with a PowerClean DNA Clean‐Up Kit (Mo Bio, Carlsbad, CA), as per the manufacturer's instructions. Samples were sequenced by The Genome Analysis Centre (TGAC, Norwich, UK) using an Illumina HiSeq 2500 platform (Illumina, San Diego, CA) in paired‐end mode, with typical read lengths 85‐150 base pairs. Samples were checked using FastQC^27^ to assure the global quality of the sequencing, and only one sample (nonpregnant, chow fed) was removed thereafter, leaving n = 6‐8 per group.

An in‐house metagenomics analysis pipeline was developed as described,^28^ introducing the removal of the repeat masking step and inclusion of the assembly step using IDBA‐UD software^29^ (Supporting Fig. S1). Methods for this are included in the Supporting Information.

### Metataxonomic Analysis (16S Ribosomal RNA Gene Sequencing)

Metataxonomics was performed by Research and Testing, Lubbock, Texas. Taxonomic identification of sequences was performed using QIIME software referencing the GreenGenes library, with statistical results analysis using STAMP and R. *P* values were adjusted for multiple comparisons using the Benjamini‐Hochberg method in R programming software.

### Metabolic Profiling

Aqueous and organic extractions were performed for cecum, cecal content, gall bladder, and liver samples as detailed in the Supporting Information. These were assessed with reversed‐phase ultra‐performance liquid chromatography tandem mass spectrometry (UPLC‐MS/MS) lipid profiling of organic extracts, hydrophilic interaction chromatography UPLC‐MS/MS profiling of aqueous extracts, and bile acid UPLC‐MS/MS profiling of combined aqueous and organic extracts.

### Data Preprocessing and Statistical Analysis of Metabolic Profiling

MS raw data were converted to netCDF format using the DataBridge tool implemented in MassLynx software (Waters Corporation, Milford, MA). Data were processed using the XCMS package in R (https://www.r-project.org/), and an output table was obtained comprising pairs of m/z_RT (mass to charge ratio _ retention time) and intensity values of the detected metabolite features in each sample. The data set was normalized to total area normalization. Multivariate data analysis was performed using the SIMCA package (v.13.0.2; Umetrics, Umea, Sweden). Principal component analysis (PCA) and orthogonal projection to latent structures discriminant analysis were used to examine UPLC‐MS data in a multivariate setting. Before model fitting, features were subjected to Pareto scaling. Two‐tailed *t* test assuming unequal variance and coefficient of variation percentage were calculated in Microsoft Office Excel 2007 (Redmond, WA). *P* values were adjusted for multiple comparisons using the Benjamini‐Hochberg method in R programming software.

### Metabolite Assignment

Metabolite identification by MS was conducted by matching accurate m/z measurements of detected chromatographic peaks to theoretical values from in‐house databases and online databases (Human metabolite database [HMDB]; http://www.hmdb.ca/), Kyoto Encyclopedia of Genes and Genomes (KEGG; http://www.genome.jp/kegg/ligand.html), LIPID MAPS (http://www.lipidmaps.org/tools/index.html), and METLIN (http://metlin.scripps.edu/)). Tandem MS fragmentation patterns were obtained for further structural elucidation. Assignment was confirmed by the comparison of retention times and MS/MS data with authentic standards (details available on request).

### Tissue mRNA Expression

Total RNA from duodenum, distal ileum, and livers of mice was extracted and quantified with real‐time PCR, as described in the Supporting Methods.

### Tissue Protein Expression

Distal ileal ASBT protein levels were measured using western blotting, as described in the Supporting Methods.

### Statistical Analysis

Statistical techniques are detailed within each section of the methodology. For all comparisons between groups, appropriate statistical tests were selected based upon the normality of data distribution, with *P* < 0.05 used as the threshold for significance following correction for multiple comparisons. Unless otherwise indicated, statistical tests were performed in R (R Foundation), Microsoft Excel (Redmond, WA) or Graphpad Prism (GraphPad Software Inc, San Diego, CA).

## Results

### Pregnancy is Associated with Reduced Enterohepatic Bile‐Acid/FXR–Mediated Signaling

**Figure 1:**
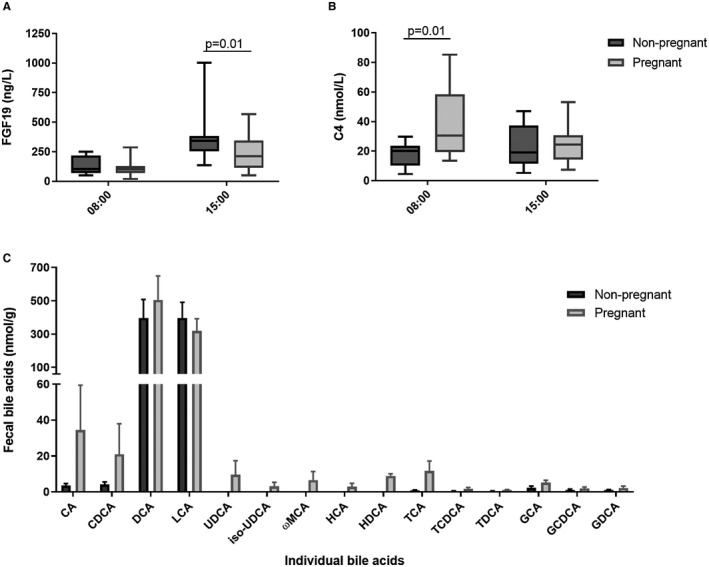
Human pregnancy reduces enterohepatic feedback, without alteration in fecal bile acids. (A) FGF19 and (B) C4 in serum of 14 nonpregnant women and 24 pregnant women with uncomplicated pregnancies while fasting (8:00 am) and 3 hours postprandial (3:00 pm), following a standardized diet. Boxes show interquartile range (IQR) with whiskers at 1.5 IQR. Groups compared with two‐way analysis of variance (ANOVA) and Tukey testing for multiple comparisons. (C) Bile acid profile of fecal samples from women outside of pregnancy (dark gray bars, n = 9) and with uncomplicated pregnancies (light gray bars, n = 14). Bars show mean ± SEM. Significance determined by two‐way ANOVA with Tukey's multiple comparison test. Abbreviations: GCA, glycocholic acid; GCDCA, glycochenodeoxycholic acid; GDCA, glycodeoxycholic acid; HCA, hyocholic acid; HDCA, hyodeoxycholic acid; TCA, taurocholic acid; TCDCA, taurochenodeoxycholic acid; TDCA, taurodeoxycholic acid; UDCA, ursodeoxycholic acid.

Fasting and peak FGF19 and C4 levels were measured in serum of women taking a standardized diet for 24 hours. Women in the third trimester of pregnancy had reduced peak FGF19, demonstrating reduced intestinal FXR signaling and enterohepatic feedback (Fig. 1A). This reduction was consistent with the increased gestational hepatic bile acid synthesis demonstrated by elevated fasting serum concentrations of C4 (Fig. 1B). Lowered FGF19 levels were not explained by intestinal FXR inhibition by PM5S, which increases in normal pregnancy and can impair hepatic FXR induction, given that CDCA‐induced FXR induction was not affected by co‐treatment with PM5S in terminal ileal explants (Supporting Fig. S2). Because bile acid species have different potencies as FXR agonists, fecal bile acids were measured. There were minimal differences between nonpregnant women and those in the third trimester of uncomplicated pregnancies (Fig. 1C). However, the majority of fecal bile acids will be deconjugated by colonic bacterial activity by the time they are excreted in feces, and therefore this result is unlikely to reflect the bile acid content of the terminal ileum, the site of maximal enterocyte FXR expression. To address this issue, cecal contents were collected from C57BL/6 pregnant mice.

### Murine Pregnancy is Characterized by Impaired Intestinal FXR Signaling, with Altered Intestinal Bile Acid Conjugation

**Figure 2:**
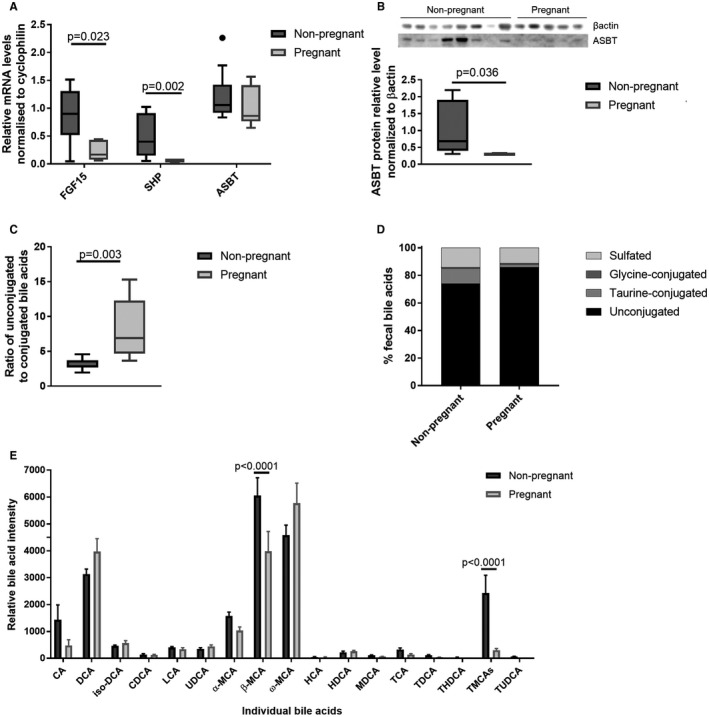
Murine pregnancy is characterized by reduced enterohepatic feedback with an altered cecal bile acid profile. (A) Expression levels of FGF15, SHP, and ASBT mRNA in murine distal ileum of nonpregnant and pregnant (day 18) mice, assessed by multiple measures of analysis of variance (ANOVA) with Tukey post‐hoc testing. Boxes show interquartile range (IQR) with whiskers at 1.5 IQR, N = 6‐8. (B) Protein levels of ASBT in murine distal ileum determined by western blotting for nonpregnant and pregnant mice (n = 8 and n = 5), normalized to βactin. Groups compared with Mann‐Whitney U test. (C) Ratio of conjugated to unconjugated bile acids in the cecal content of nonpregnant and pregnant mice. Boxes show IQR with whiskers at 1.5 IQR, N = 6‐8. (D) Distribution of cecal bile acids by conjugation in the cecal content of nonpregnant and pregnant mice. N = 6‐8. (E) Relative quantification of individual bile acids present in cecal content of nonpregnant and pregnant mice. Assessed by Student *t* test, with Benjamini‐Hochberg correction, results show mean + SEM, N = 6‐8. Abbreviations: HCA, hyocholic acid; HDCA, hyodeoxycholic acid; MDCA, murideoxycholic acid; TCA, taurocholic acid; TDCA, taurodeoxycholic acid; THDCA, taurohyodeoxycholic acid; TUDCA, tauroursodeoxycholic acid; UDCA, ursodeoxycholic acid.

To determine the influence of pregnancy on intestinal tissues, terminal ileal and cecal samples from C57BL/6 mice were examined. Terminal ileal FXR induction of FGF15 and SHP was reduced on day 18 of murine pregnancy (Fig. 2A). Whereas expression of ASBT mRNA did not differ significantly in pregnancy, despite its usual repression by SHP (Fig. 2A), ASBT protein levels were significantly reduced (Fig. 2B). Consistent with the human explant models, PM5S did not repress terminal ileal FXR induction in mice that received PM5S gavage compared to vehicle (Supporting Fig. S3). Given the gestational reduction in terminal ileal FXR induction, we determined hepatic mRNA expression of genes of relevance to bile acid homeostasis, to establish whether these intestinal signals contribute to hypercholanemia (Supporting Fig. S4A). There were gestational reductions in mRNA expression of Fxr target genes as has been described.^4^ Intriguingly, there was reduced transcription of cytochrome P450 family 7 subfamily A member 1 (*Cyp7a1*) at day 18 of pregnancy, a result that is not consistent with reduced bile acid synthesis. Because murine pregnancy is associated with hypercholanemia with advancing gestation, which could repress *Cyp7a1* at the hepatocyte,^3^ we quantified hepatic *Cyp7a1* expression in mice throughout pregnancy and demonstrated a significant increase at day 7 of pregnancy, which fell by day 14 (Supporting Fig. S4B).

Targeted UPLC‐MS/MS evaluation of cecal content demonstrated lower conjugated and sulfated bile acids in pregnancy (Fig. 2C,D; Supporting Table S2), with proportionately more secondary (DCA, ω‐MCA) than primary bile acids (CA, α‐MCA, β‐MCA) than in age‐matched nonpregnant mice (Fig. 2E). The reduction in conjugated bile acids in the cecum in pregnancy did not result from lower hepatic bile acid conjugation, given that the bile acid profiles of liver and gall bladder did not reveal altered conjugation proportions in pregnancy (Supporting Fig. S5).

### The Cecal Microbiome of Murine Pregnancy has Elevated *Bacteroidetes*, with Enhanced Bile Salt Hydrolase Genes and Bile Salt Deconjugation

**Figure 3:**
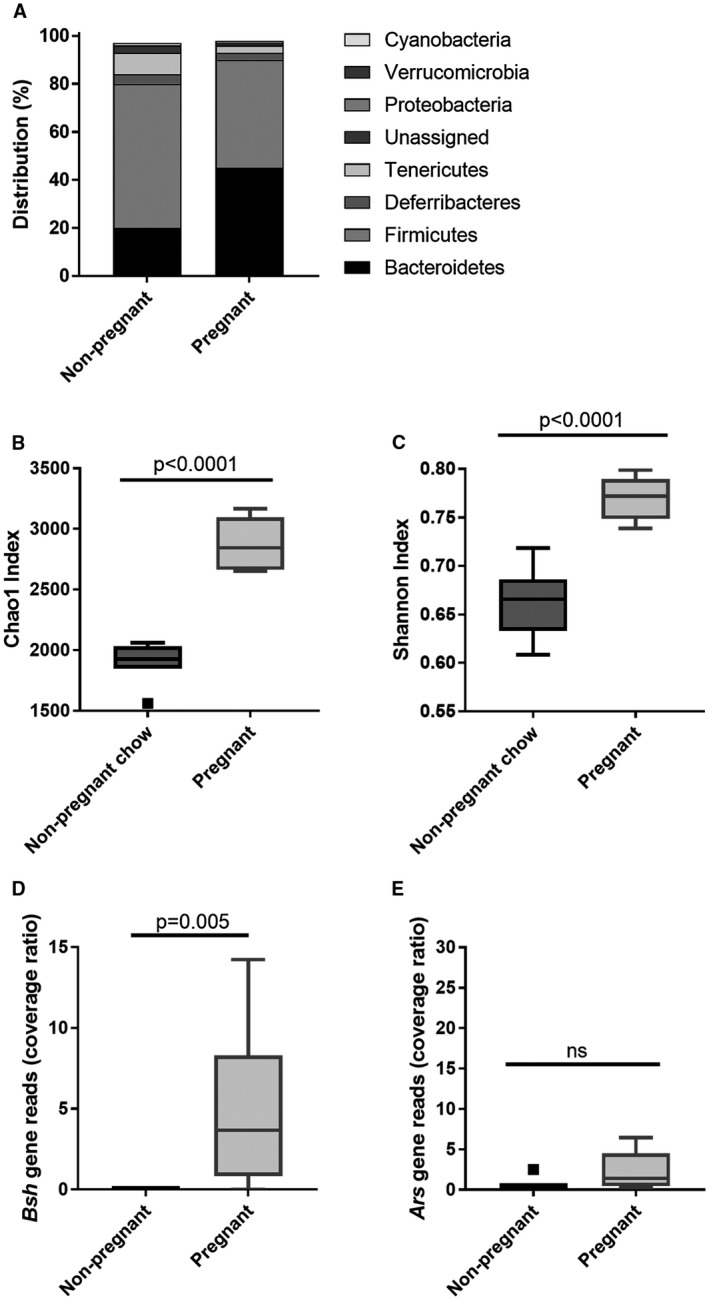
Pregnancy alters the murine cecal microbiota and microbiome. (A) Distribution of cecal microbiota by phylum level for nonpregnant and pregnant mice. N = 6‐8. (B) Chao 1 richness and (C) Shannon evenness of microbial communities for nonpregnant and pregnant mice. Box‐whisker plots demonstrate median (thick line) and interquartile range (IQR) with whiskers at 1.5 IQR. Differences between groups determined by Kruskal‐Wallis univariate analysis. N = 6‐8 throughout. (D)* Bsh* and (E) aryl sulfatase (*Ars*) gene reads in the cecal microbiome of nonpregnant and pregnant mice. Difference between groups determined by two‐tailed *t* test. N = 6‐8 throughout. Abbreviation: ns, not significant (*P* ≥ 0.05).

Given that the cecal bile acid composition was likely altered by the intestinal bacterial modification, whole shotgun genome metagenomic sequencing was used to characterize the effect of pregnancy on the murine cecal microbiota. Pregnancy was associated with a higher ratio of *Bacteroidetes* to *Firmicutes*, with increased richness and diversity (Fig. 3A‐C; Supporting Table S3). Targeted metagenomics confirmed the pregnant microbiome encoded bile salt hydrolase (*Bsh*), which deconjugates bile acids; further analysis revealed this to be encoded by the genome of bacteria of the *Bacteroidetes* phylum (Fig. 3D). Similarly, the microbiome of pregnant mice included genes for aryl sulfatases, also *Bacteroidetes* derived, which were not present outside pregnancy (Fig. 3E). 7‐α‐dehydroxylase sequences from the Uniprot library were not identified in the microbiome of either group. However, none of these sequences originates from *Bacteroidetes*, which are likely to have contributed to the production of secondary bile acids.

### The Gestational Murine Cecal Metabonome Markedly Differs from Nonpregnant Mice, with Lower Taurine Secondary to its Bacterial Metabolism

**Figure 4:**
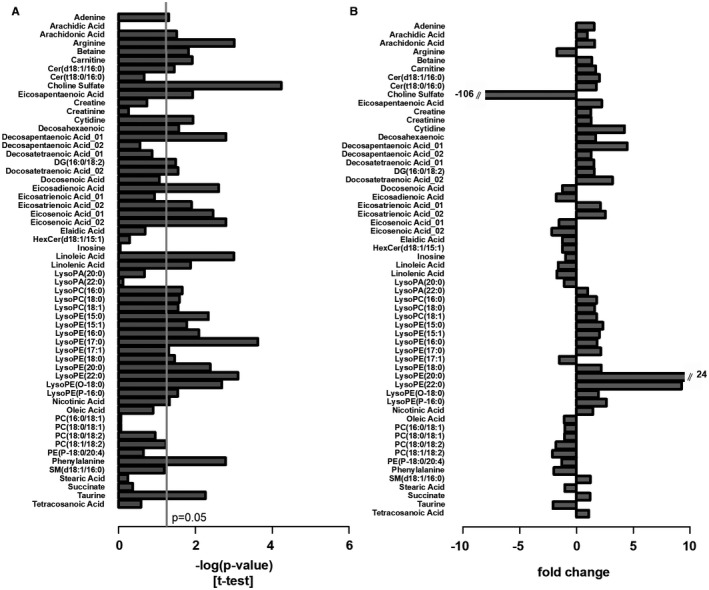
Pregnancy alters the murine cecal metabonome. Metabolite changes in the cecal content between nonpregnant and pregnant mice. (A) Significance of metabolite changes, log (*P* value) of *t* test (two‐tailed; assuming unequal variance) and (B) fold change for the same metabolites. *P* values adjusted for multiple comparisons using the Benjamini‐Hochberg method. N = 6‐8. Abbreviations: Cer, ceramide; DG, diacylglycerol; HexCer, hexosylceramide; PA, phosphatidic acid; PE, phosphatidylethanolamine; PC, phosphatidylcholine; SM, sphingomyelin. N = 6‐8.

Given the multiple metabolic actions of intestinal bacteria, untargeted UPLC‐MS/MS was performed to identify metabolites that were significantly altered in the cecal content of pregnant mice (Fig. 4A,B; summary of model characteristics in Supporting Table S4). Taurine was significantly reduced in pregnancy, despite the increased deconjugation inferred by cecal bile acid profiles and bacterial BSH. The cecal microbiota in pregnancy was enriched with sulfur‐utilizing bacteria, such as the *Proteobacteria Bilophila* (Supporting Table S3); these likely utilized the liberated taurine from *Bacteroidetes* BSH bile acid deconjugation, or sulfur following aryl sulfatase activity, as a growth substrate. Untargeted metabonomics did not detect any differences in known FXR ligands, although the RXR ligands, docosahexaenoic acid, arachidonic acid, and docosapentaenoic acid,^30^ were increased in pregnancy.

### CA Feeding of Mice Reversed the Gestational Impairment of FXR‐Mediated Enterohepatic Feedback

**Figure 5:**
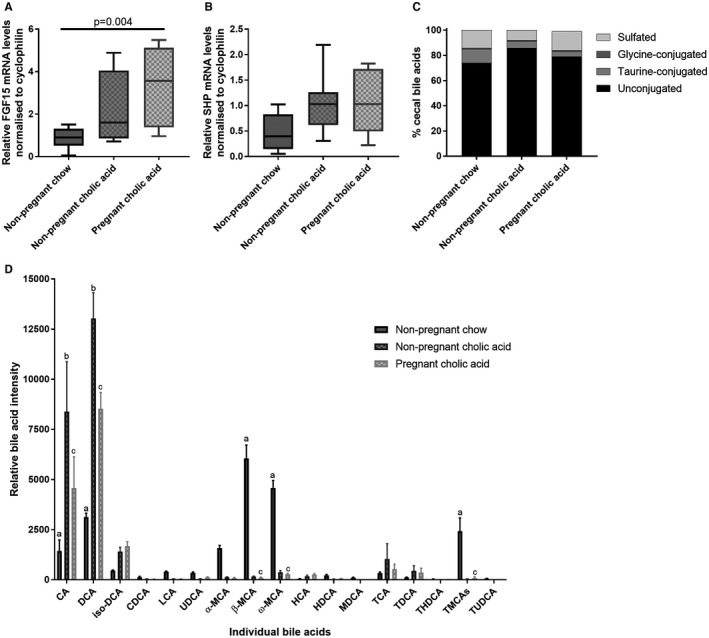
CA supplementation enhances enterohepatic feedback and alters the cecal bile acid profile. (A) Expression levels of FGF15 and (B) SHP mRNA in murine distal ileum of nonpregnant chow‐ and CA‐fed mice and pregnant CA‐fed mice, assessed by multiple measures of analysis of variance with Tukey post‐hoc testing. Boxes show interquartile range (IQR) with whiskers at 1.5 IQR, N = 6‐8. (C) Distribution of cecal bile acids by conjugation in the cecal content of nonpregnant chow‐ and CA‐fed and pregnant CA‐fed mice. N = 6‐8. (D) Relative quantification of individual bile acids present in cecal content of nonpregnant chow‐ and CA‐fed mice and pregnant CA‐fed mice. Assessed by two‐tailed *t* test, with Benjamini‐Hochberg correction; bars show mean + SEM, N = 6‐8. Significance determined by *P* < 0.05; a: nonpregnant chow‐ versus nonpregnant CA‐fed mice; b: nonpregnant CA‐fed versus pregnant CA‐fed mice; c: nonpregnant chow‐fed versus pregnant CA‐fed mice. Abbreviations: HCA, hyocholic acid; HDCA, hyodeoxycholic acid; MDCA, murideoxycholic acid; TCA, taurocholic acid; TDCA, taurodeoxycholic acid; THDCA, taurohyodeoxycholic acid; TUDCA, tauroursodeoxycholic acid; UDCA, ursodeoxycholic acid.

Given that these results suggest that the impairment in FXR‐mediated enterohepatic feedback in pregnancy is attributed to altered intestinal bile acid species availability, with reduced terminal ileal uptake attributed to the lower ASBT affinity of unconjugated bile acids, we sought to identify whether this could be overcome with an increased intestinal bile acid load. A diet supplemented with 0.5% CA was fed to pregnant C57BL/6 mice and to age‐matched nonpregnant mice. CA‐supplemented mice had increased terminal ileal FXR induction, with elevated FGF15 mRNA expression (Fig. 5A). Similarly, CA‐fed mice did not have the suppression of SHP (Fig. 5B) that is observed in normal gestation. Supplementation with CA altered the cecal bile acid composition, with increased CA and its bacterially derived metabolite, DCA (Fig. 5C,D). CA‐fed mice had very low cecal MCA species; these are dependent upon murine hepatic synthesis, confirming that CA supplementation overcame the gestational impairment in FXR‐mediated enterohepatic feedback.

### CA Supplementation had Similar Effects on the Cecal Metabonome to Pregnancy

**Figure 6:**
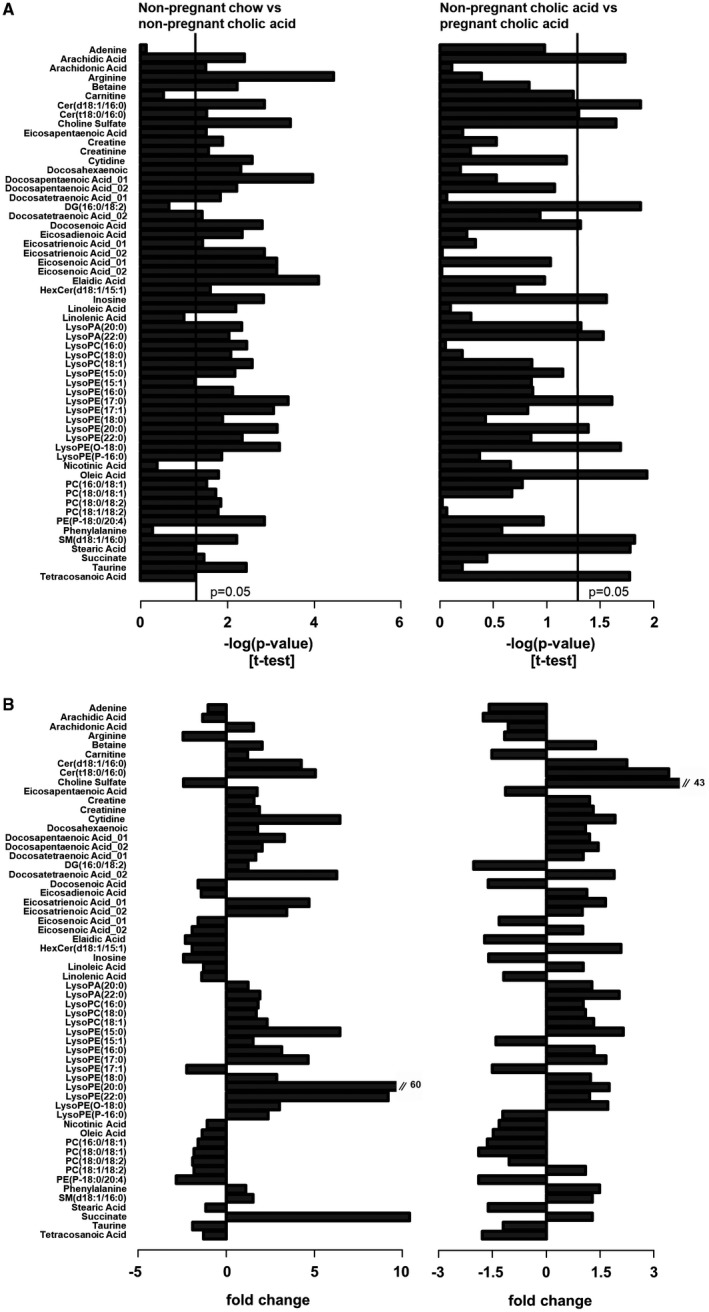
CA dietary supplementation alters the murine cecal metabonome. Metabolite changes in the cecal content between, from left to right, nonpregnant chow‐ and nonpregnant CA‐fed mice, and nonpregnant CA‐ and pregnant CA‐fed mice. (A) Significance of metabolite changes, log (*P* value) of *t* test (two‐tailed; assuming unequal variance) and (B) fold change for the same metabolites. *P* values adjusted for multiple comparisons using the Benjamini‐Hochberg method. N = 6‐8. Abbreviations: Cer, ceramide; DG, diacylglycerol; HexCer, hexosylceramide; PA, phosphatidic acid; PE, phosphatidylethanolamine; PC, phosphatidylcholine; SM, sphingomyelin. N = 6‐8.

To support the hypothesis that intestinal induction of FXR resulted from the altered bile acid profile, untargeted metabonomics was performed. CA supplementation resulted in very similar metabolite profiles to pregnancy (Figs. 4 and 6).

### The Gestational Alterations to the Cecal Microbiota are Enhanced in CA‐Supplemented Mice

**Figure 7:**
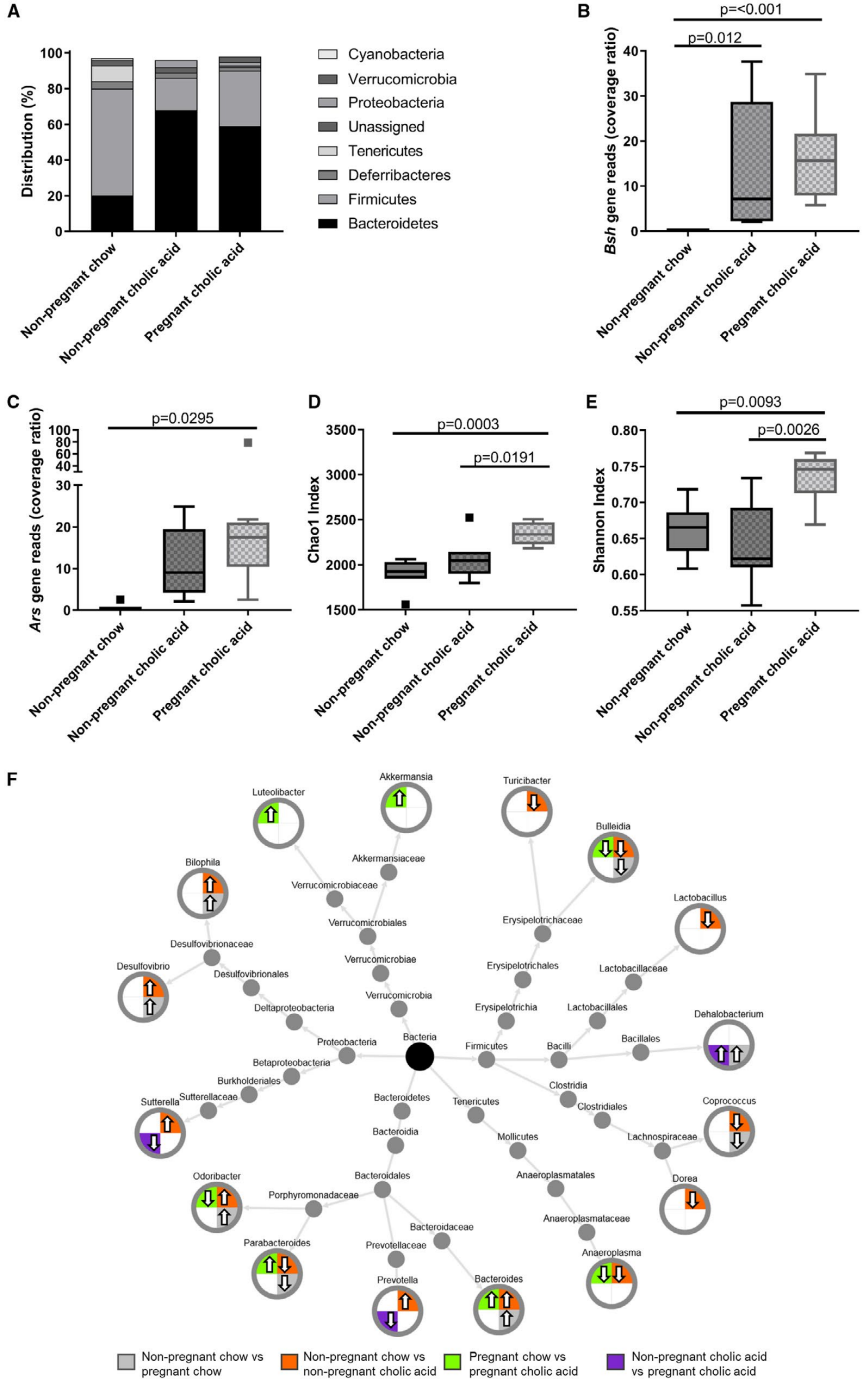
CA dietary supplementation alters the murine cecal microbiota in similar ways to pregnancy. (A) Distribution of cecal microbiota by phylum level for nonpregnant chow‐ and CA‐fed mice, and pregnant CA‐fed mice. N = 6‐8. (B) *Bsh* and (C) aryl sulfatase (*Ars*) gene reads in the cecal microbiome of nonpregnant and pregnant mice. Box‐whisker plots demonstrate median (thick line) and interquartile range (IQR), with whiskers at 1.5 IQR. Differences between groups determined by one‐way analysis of variance with Tukey's multiple comparison test. N = 6‐8. (D) Chao 1 richness and (E) Shannon evenness of microbial communities for nonpregnant and pregnant mice. Box‐whisker plots demonstrate median (thick line) and IQR, with whiskers at 1.5 IQR. Differences between groups determined by Kruskal‐Wallis univariate analysis. N = 6‐8 throughout. (F) Taxonomic tree of microbes identified at genus level found to significantly differ after separate comparisons of nonpregnant chow‐ versus pregnant chow‐fed (gray), nonpregnant chow‐ versus nonpregnant CA‐supplemented diet (orange), pregnant chow‐ versus pregnant CA‐supplemented diet (green), and nonpregnant CA‐ versus pregnant CA‐supplemented diets (purple). Arrow direction indicates relative abundancy in the second of the comparator groups. Significance defined as *P* < 0.05 by White's nonparametric *t* test with Benjamini‐Hochberg correction.

**Figure 8:**
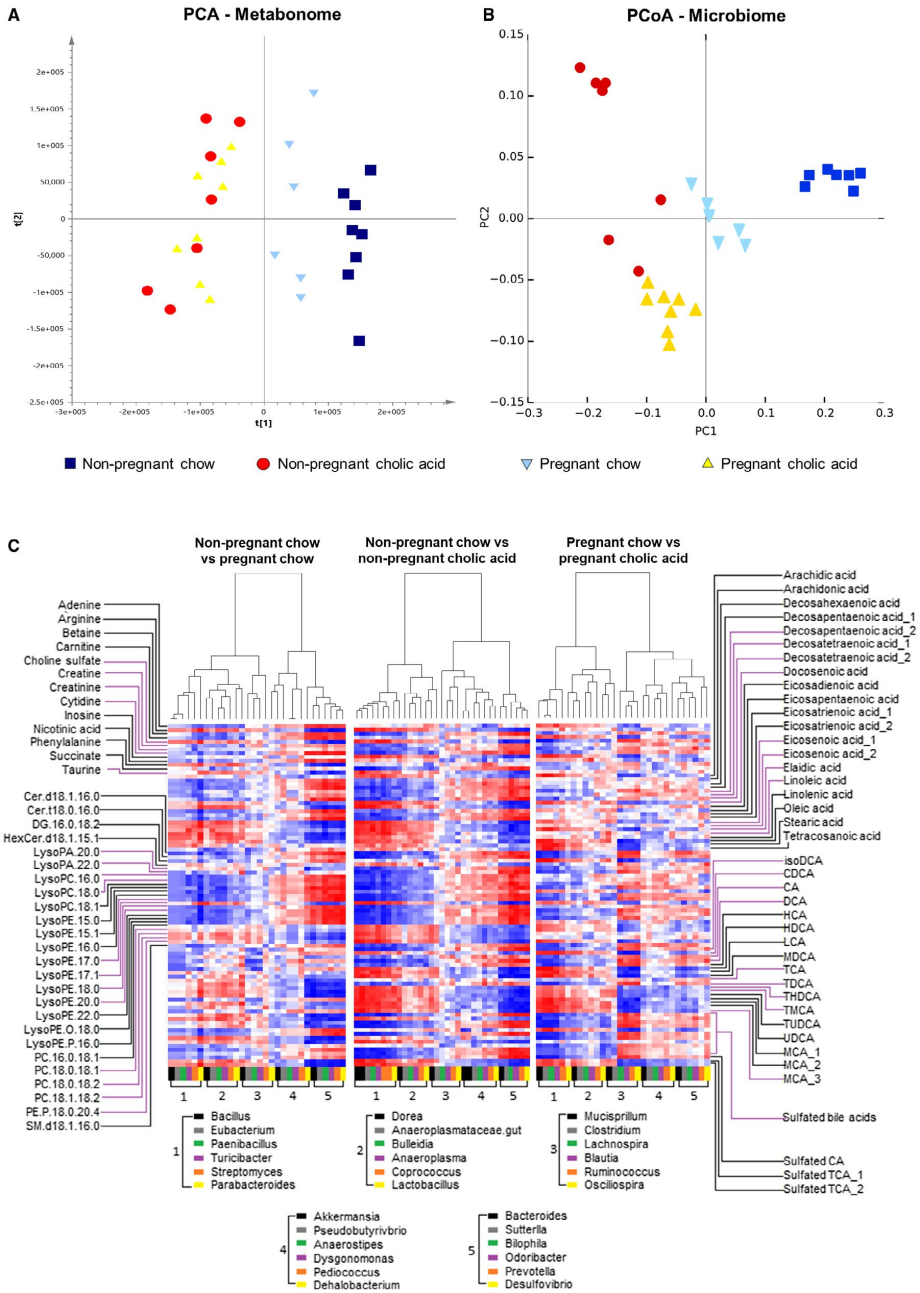
Comparison between metabonome and microbiome of mouse cecal content by diet and pregnancy status. (A) Scores plot of PCA of the cecal content extracts generated from the UPLC‐MS analysis (dark blue, nonpregnant chow‐fed; light blue, pregnant chow‐fed; red, nonpregnant CA‐supplemented diet; yellow, pregnant CA‐supplemented diet). PC1 explains 27% variance of the data set, and PC2 explains 13.6%. (B) Scores plot of principal coordinates analysis (PCoA) of the cecal content from the microbiome weighted Unifrac analysis (dark blue, nonpregnant chow‐fed; light blue, pregnant chow‐fed; red, nonpregnant CA‐supplemented diet; yellow, pregnant CA‐supplemented diet). PC1 explains 64% variance of the data set, and PC2 explains 12.6%. (C) Correlation matrix (Spearman correlation) of metabolites (y‐axis) and microbes (x‐axis) found to be significantly different after separate comparisons of nonpregnant chow‐ versus pregnant chow‐fed, nonpregnant chow‐ versus nonpregnant CA‐fed, and pregnant chow‐ versus pregnant CA‐fed mice. N = 6‐8 throughout.

CA supplementation enriched the gut microbiota with *Bacteroidetes* and *Proteobacteria*, with reduced *Firmicutes*, similar to the changes observed in pregnancy (Fig. 7), although the increased richness and diversity was only demonstrated in the pregnant CA‐fed mice. Whereas pregnancy ameliorated the CA‐feeding enhancement of *Sutterella* and *Prevotella*, the pregnancy‐associated increase of *Dehalobacterium* was observed irrespective of CA supplementation. Additionally, CA supplementation enhanced bacteria encoding genes for BSH expression. Ordination methods of comparing the cecal metabonome and microbiota demonstrated that CA feeding enhances the gestational changes observed (Fig. 8A,B). A heatmap correlating individual microbial and metabolite levels for the different murine groups demonstrated that the alterations observed were particularly similar for both gestational and CA‐feeding effects (Fig. 8C), demonstrating the critical inter‐relationship between the gut microbial profile and metabolite composition. In both groups, the taurine‐conjugated bile acids were negatively correlated with the microbes that became more abundant in pregnancy (*Bacteroides*,* Odoribacter*,* Prevotella*,* Bilophila*, and *Desulfovibrio*), and they had a positive correlation with those that were reduced (*Parabacteroides* and *Bulleidia*). Taurine showed similar correlations with the microbiota to the taurine‐conjugated bile acids, and there was an increased abundance of microbes that are known to utilize taurine for energy in the cecal content of pregnant and CA‐fed animals, such as *Bilophila* and *Desulfovibrio* spp. Of the phospholipids, lysophosphatidylethanolamines (LPEs) and lysophosphatidylcholines (LPCs) positively correlated with the microbes that were increased in pregnancy and CA feeding (e.g., *Bacteroides*, *Oridobacter*,* Prevotella*,* Bilophila*, and *Desulfovibrio*) and negatively correlated with those that were reduced (*Bulleidia*, *Coprococcus*, and *Parabacteroides*). The only exception was LPE (17:1), which had the reverse correlation. For pregnant CA‐fed mice, the associations between metabolites and microbiota more closely reflected those observed for CA‐fed mice than pregnancy alone (e.g., for inosine, docosenoic acid, tetracosanoic acid, diacylglycerol, and the bile acids), although this was not universal (e.g., taurine).

Using the metagenomic functional analysis and metabolite results, the effects of pregnancy, CA feeding, and CA feeding in pregnancy on metabolic pathways of the mice (host) and microbiota (colonizer) were determined. Results were strikingly similar for the effects of pregnancy and CA feeding, especially with respect to the predicted induction of host hepatotoxic pathways (Supporting Table S5). Overlap in the pregnancy/CA‐fed comparators is illustrated in the significantly altered toxicology functions (Supporting Fig. S6), diseases, and biofunction pathways and canonical pathways (Supporting Figs. S7 and S8). Specifically, for normal pregnancy, the 10 most significantly altered toxicological functions included six functions implicated in liver damage or liver steatosis, and eight such pathways were altered in CA‐fed animals, the majority of which were the same as in normal gestation (Supporting Fig. S6). Pathway analyses further revealed the cecal microbiome of pregnancy and CA feeding in mice to have significantly increased abundance of genes encoding components of the glycine cleavage system (Supporting Fig. S9), consistent with utilization of glycine as an additional energy source, likely available following the additional deconjugation of glycine‐conjugated bile acids.

## Discussion

Our results demonstrate that normal pregnancy is associated with impaired FXR‐mediated enterohepatic feedback and elevated hepatic bile acid synthesis, which is consistent with the elevation in serum primary bile acids (CA > CDCA) observed in human pregnancy.^1^ We concluded that this impairment results from an altered intestinal bile acid composition secondary to gestational changes in the gut microbiota, with enhanced *Bacteroidetes*‐mediated bile acid deconjugation in combination with reduced terminal ileal bile acid uptake in pregnancy secondary to lower ASBT protein levels. Direct signaling from the gut bacteria to FXR or other alterations in the gut metabonome are unlikely to cause this impairment in enterohepatic feedback, given that CA dietary supplementation resulted in a very similar gut microbiome and metabolome to pregnancy, yet resulted in induction of enterohepatic feedback. Pregnant and CA‐supplemented mice had increased cecal ratios of *Bacteroidetes* to *Firmicutes*, with additional enrichment of sulfur‐reducing bacteria. Bile salt hydrolase was exclusively detected in the genome of the *Bacteroidetes* present in these animals, and these mice had proportionately more cecal unconjugated bile acids.

The elevation in serum bile acids in pregnancy has previously been attributed to reduced enterohepatic cycling,^31^ consistent with reduced FXR function and bile salt export pump induction^4^, ^7^; our demonstration of elevated fasting C4, in particular, supports altered regulation of bile acid synthesis in pregnancy, likely secondary to reduced FXR function and resultant lowering of SHP‐mediated impairment of CYP7A1. Whereas we demonstrated reduced *Cyp7a1* mRNA expression at day 18, the increased transcription at day 7 is consistent with hepatic FXR inhibition; protein quantification and activity assays would be required to confirm this mechanism of increased gestational bile acid synthesis. Herein, we provide an additional explanation, that increased hepatic bile acid synthesis is secondary to reduced enterohepatic feedback, which is consistent with the previous murine study of Moscowitz et al.^32^ A limitation of our study was that we did not measure the complete bile acid pool size and total bile acid excretion, to confirm that increased bile acid synthesis was not compensatory to increased fecal bile acid loss.

Alterations in the intestinal bile acid composition, resulting from bacterial bile acid modification, could impact enterocyte FXR induction in two ways: by impairing bile acid uptake by the enterocyte and/or by changing the proportion of agonistic bile acid ligands compared with the antagonists. ASBT binding of bile acids is thought to be the rate‐limiting step in the uptake of bile acids from the lumen to terminal ileal enterocytes, and ASBT preferentially binds conjugated bile acids (reviewed in a previous work^33^). Thus, we hypothesize that the proportionate reduction in conjugated bile acids in pregnancy, secondary to bacterial activity, reduces the amount of bile acids absorbed in the terminal ileum, the predominant site of FGF19/15 synthesis. Whereas there was no reduction in ASBT mRNA induction in pregnancy, ASBT protein levels were reduced in the distal ileum, which likely contributed further to the reduced intestinal FXR induction.

Established ligand potencies of the bile acids present in the pregnant cecal content are consistent with altered FXR induction as a consequence of the different balance of agonistic bile acids, such as CDCA and DCA species,^17^ with antagonistic bile acids, such as the tauromuricholic acids (TMCAs).^15^ The profile of cecal bile acids in pregnant mice did not suggest that these mice would have associated impairment of intestinal FXR function, given that there were significant reductions in the antagonistic species.

The similarities in the intestinal metabonome and microbiota between the pregnant and CA‐fed mice were surprising, given the markedly different bile acid loads to the intestine. Our findings are in contrast to those of CA dietary supplementation in rats, where increased cecal CA and DCA induced expansion of the *Firmicutes*, particularly the Clostridia class.^34^ This may have been attributed to the increased lysophospholipids (LPE/LPC) in the cecal content; these lysophospholipids are bactericidal to Gram‐negative bacteria, particularly anaerobes such as Clostridia.^35^, ^36^ Given the structural similarity of steroid hormones to bile acids, the gestational increases in estrogens, progestogens, and corticosteroids, present in bile (reviewed by Begley et al.^37^), may provide similar bacterial selection pressures to those of bile acid feeding. Indeed, steroid hormones, such as 17α‐hydroxyprogesterone, estradiol, and progesterone, are both inhibitory and stimulatory to growth of different bacteria.^38^, ^39^, ^40^

In summary, we have demonstrated alterations in the intestinal microbiome and metabonome in pregnancy that could impair enterohepatic feedback on bile acid synthesis and contribute to the hypercholanemia of pregnancy. Particularly given that the secondary bile acids, DCA and lithocholic acid (LCA), are also potent ligands for the TGR5 receptor at enteroendocrine L cells, the altered microbial modification of bile acids in pregnancy may explain the increasing levels of GLP1 with advancing gestation.^41^ Future studies are needed to establish precisely how gestational signals are capable of dramatically impacting the gut microbiome during pregnancy, and whether these gestational signals can be manipulated to enhance beneficial metabolic changes for the pregnancy. This understanding will be of importance for the treatment of such gestational metabolic disorders as intrahepatic cholestasis of pregnancy and gestational diabetes mellitus.
