# Supplementary material for: Enhanced Microbial Bile Acid Deconjugation and Impaired Ileal Uptake in Pregnancy Repress Intestinal Regulation of Bile Acid Synthesis
Source: Hepatology. 2019 May 21;70(1):276–93. doi: 10.1002/hep.30661 (PMC6619257; doi:10.1002/hep.30661)
Supplement: Supplementary file 1 [file HEP-70-276-s001.pdf]

Supplementary methods

Terminal ileal explants

Terminal ileal biopsies were obtained from non-pregnant individuals undergoing routine colonoscopy, as previously described(1). Explants (2 per well) were cultured with the following treatments (n=6-8): culture medium, 50µmol/L chenodeoxycholic acid (CDCA), 50µmol/L epiallopregnanolone sulfate (PM5S), and 50µmol/L CDCA + 50µmol/L PM5S for 6hours, following which they were stored in RNAlater at -80°C.

Murine progesterone sulfate administration

C57BL/6 mice were administered either 200µL of 20% cyclodextrin (vehicle) or 500mg/kg PM5S by oral gavage (n=5 per group, non-pregnant), and terminal ileum harvested for measurement of mRNA expression.

Metagenomics analysis

The code was integrated in a distributed layer for executing in parallel. Briefly, the samples were paired-end joined using SeqPrep, trimmed and filtered using Trimmomatic(2). PhiX and murine sequences were removed using Bowtie2(3) and duplicates were removed using usearch(4). 16S rRNA gene sequences were extracted using rRNASelector(5) and taxonomically analyzed using QIIME(6) with the GreenGenes database(7) as the reference to which they were classified; remaining sequence data was assembled using IDBA-UD. Coding domain sequences (CDS) were predicted using FragGeneScan(8) and PFAM database was queried using these CDSs using Interproscan(9).

A bespoke Python script (available on request) was employed for re-formatting the output and adapting it to different statistical analysis software. White's non-parametric t-test was performed using Statistical Analysis of Metagenomic Profiles (STAMP)(10) and all p-values were corrected using Benjamini-Hochberg method. PCoA was calculated using weighted Unifrac distance method. Taxonomic tree was created using an internal development for visualization of taxonomic differences between experimental groups.

ShortBRED (https://huttenhower.sph.harvard.edu/shortbred, Kaminski et al., in progress,) software was used for the functional targeted analyses of arylsulfatase and 7-α-dehydroxylase genes using a specific selection of sequences in Uniprot database and for the BSH analysis using an internal database of BSH sequences.

The microbial pathway ranking was created using the cross-referenced PFAM – EC KEGG output of Interproscan software and the following ranking criteria: Large pathways (more than 20 enzymes involved) were considered to be changed i.e. depleted or over-represented if a minimum of 10% of the involved enzymes were changed, in either direction viz increased or reduced in abundance. Small pathways (less than 20 enzymes involved) were considered to be changed if a minimum of 2 enzymes were changed. A list of KEGG pathways considered changed using these criteria is presented in Supplementary Table 5.

The integration of host and microbial disturbed pathways was performed using QIAGEN's Ingenuity Pathway Analysis (IPA®, QIAGEN Redwood City, www.qiagen.com/ingenuity).

Sample preparation for metabolic profiling

The extraction protocol was adapted from Want et al., 2013(11).

Aqueous extraction. Cecum, cecal content and liver samples (50 mg) were placed into separate bead beating tubes (VWR, UK), which were preloaded with 1mm zirconium beads (BioSpec USA). To obtain the aqueous extract, 1.2mL of chilled methanol/ water 1:1 solution (LC-MS grade, Fisher) was added to each tube after randomization of the samples. Samples were subsequently loaded onto a bead beater (Bertin Technologies). Three blank samples were also prepared containing only the mixture of solvents and the zirconium beads in order to identify contaminants introduced by the solvents and tubes. The bead beater vibrated at 6500Hz for 40 s and 2 + 2 cycles were performed separated by freezing of the samples on dry ice for 5 minutes between the cycles. Then samples were centrifuged (Eppendorf, Centrifuge 5417R, Germany) at 20,000 x g for 20 minutes at 4°C. 1ml of supernatant was obtained from each sample and further divided into 2 aliquots of 500 µl for HILIC and bile acid UPLC-MS/MS profiling. Extraction was followed by drying the samples in a vacuum concentrator for 3 hours at 45°C in V-AQ mode (Eppendorf Concentrator Plus). Samples were stored at -40°C until analysis.

Organic extraction. Pre-chilled 1.2mL solution of 3:1 dichloromethane/ methanol (dichloromethane: HPLC grade, Sigma-Aldrich) was added to the residual pellet. Samples were frozen on dry ice and re-loaded into the bead beater following the same operating program but only for two cycles. Samples were centrifuged at 20,000 x g for 20 min at 4°C, and 2 aliquots of 400µL organic phase supernatant were placed into glass vials for lipid and bile acid UPLC-MS/MS profiling. Samples were evaporated at room temperature in an extractor hood and stored at -40°C until analysis.

Metabolic profiling analysis

RP-UPLC-MS/MS Lipid Profiling of Organic Extracts.

Adapted from Vorkas et al., 2015(12) and Spagou et al., 2011(13).

The organic extracts of cecum and cecal content samples were reconstituted in a mixture of isopropanol/ acetonitrile/ water (2:1:1, 250µL), vortexed for 30s, sonicated for 5min and vortexed for 30s, followed by centrifugation at 20,000xg for 30min at 4 °C. Supernatant was transferred into glass inserts in the LC-MS vials. A Quality Control (QC) sample was prepared with 50µL of each sample, to assess analytical reproducibility. The column was conditioned by injecting the QC pooled sample, several times, until data showed adequate stability. The QC sample was then injected every 6 samples to monitor the instrument's performance. The analytical run was completed by the analysis of extraction and solvent blank samples.

Lipid profiling was performed on an Acquity UPLC system (Waters Corp, USA) coupled to a XEVO G2 QTof Mass Spectrometry system (Waters MS Technologies, UK). Chromatography was performed using an Acquity UPLC CSH C18 2.1x100mm, 1.7um and column (Waters Corporation, USA) was held at 55°C. Separation was achieved using gradient elution with 0.1% (v/v) formic acid in acetonitrile/water (60:40) (A) and 0.1% (v/v) in isopropanol/acetonitrile (B) (90:10) at a flow rate of 0.4 mL/min. In both mobile phases ammonium formate (LC-MS grade, Fluka, USA) was diluted to 10mM. Starting conditions were 60%A and 40%B for 2min, changing linearly to 43%B over 2min, to 50%B within 0.1min and to 54%B over 10min, when it was changed to 70%B within 0.1min and to

99%B over 6min. The solvent composition then returned to starting conditions over 0.1min, followed by re-equilibration for 2min prior to the next injection. Mass spectrometry was performed using electrospray in both positive and negative ESI ionization modes. The capillary voltage was 1.5kV, sampling and extraction cone voltages were 20V and 4V respectively, desolvation temperature was 600 °C, and source temperature was 120 °C. The cone gas flow rate was 50L/h, and desolvation gas flow rate was 1000L/h. The MS was operated in sensitivity mode with a scan time of 0.2s. For mass accuracy, a LockSpray interface was used with a 5ng/L leucine enkephalin (555.2645 amu) solution (50/50 ACN/H2O with 0.1% v/v formic acid) at 15µL/min was used as the lock mass. Data were collected in centroid mode with a scan range of 50−2000 m/z, with lockmass scans collected every 30s and averaged over 4 scans to perform mass correction. Injection volumes of 4µL and 15µL were used for positive and negative ionization modes respectively. The auto-sampler was set at 4°C.

HILIC UPLC-MS/MS Profiling of Aqueous Extracts.

Adapted from Vorkas et al., 2015(12) and Spagou et al., 2011(13).

The aqueous extracts of cecum, cecal content and liver samples were reconstituted in a mixture of acetonitrile/water (1:1, 170µL), vortexed for 30s, sonicated for 5min and vortexed for 30s, followed by centrifugation at 20,000xg for 30min at 4 °C. The supernatant (100µL) from each sample was transferred into glass inserts in the LC-MS vials. A QC sample was prepared by aliquoting 50µL of each sample and the same QC strategy was used as above. The analytical run was completed by the analysis of extraction and solvent blank samples.

HILIC-UPLC-MS/MS analysis was performed using an identical Acquity UPLC system (Waters Corp, USA) coupled to a XEVO G2 QTof Mass Spectrometry system (Waters MS Technologies, UK) as the one used for lipid profiling of organic extracts. Column temperature was set at 40°C. Mobile phase A consisted of ACN/water (95:5) and mobile phase B ACN/water (50:50). In both solutions ammonium acetate was diluted to 10mM and formic acid to 0.1%. Separation was achieved using gradient elution: starting conditions were 99%A for 2min with flow rate 0.4ml/min, changing linearly to 55% B over the next 8min, and then to 99%B within 1 min, at which it was kept for 2min. Subsequently, the solvent composition returned to starting conditions over 0.1min, followed by re- equilibration for 9 min with increasing flow rate (up to 0.9ml/min) prior to the next injection. Mass spectrometry was performed using electrospray in both positive and negative ESI ionization modes. The capillary voltage was 1.5kV, sampling and extraction cone voltages were 30V and 4V respectively, desolvation temperature was 600 °C, and source temperature was 120 °C. The cone gas flow rate was 50L/h, and desolvation gas flow rate was 1000L/h. The MS was operated in sensitivity mode with a scan time of 0.2s. For mass accuracy, a LockSpray interface was used with a 5ng/L leucine enkephalin (555.2645 amu) solution (50/50 ACN/H2O with 0.1% v/v formic acid) at 15µL/min was used as the lock mass. Data were collected in centroid mode with a scan range of 50−1200 m/z, with lockmass scans collected every 30s and averaged over 4 scans to perform mass correction. Injection volume of 2µL was used for both positive and negative ionization modes. The auto-sampler was set at 4°C.

Bile Acid UPLC-MS/MS profiling of combined Aqueous and Organic Extracts.

Adapted from Sarafian et al., 2015(14).

The remaining second dried aliquots of aqueous and organic extracts of cecum and cecal content samples were combined prior to analysis. The aqueous extracts were reconstituted in a mixture of

propanol/ water (1:1, 150µL), vortexed for 30s, sonicated for 5min and vortexed for 30s. The supernatant was transferred into the dried organic extracts, vortexed for 30s, sonicated for 5min and vortexed for 30s, followed by centrifugation at 20,000xg for 30 min at 4 °C. 100µL supernatant from each sample was transferred into glass inserts in the LC-MS vials. A QC sample was prepared by aliquoting 40µL of each sample and the same QC strategy was used as above. The analytical run was completed by the analysis of extraction and solvent blank samples. Bile acid UPLC-MS/MS analysis was performed using an identical Acquity UPLC system (Waters Corp, USA) coupled to a XEVO G2 QTof Mass Spectrometry system (Waters MS Technologies, UK) as the one used above. An ACQUITY BEH C8 column (1.7µm, 100mm × 2.1mm) was used at an operating temperature of 60 °C. The mobile phase solvent A consisted of a volumetric preparation of 100mL of acetonitrile added to 1L of ultrapure water, with a final additive concentration of 1mM ammonium acetate and pH adjusted to 4.15 with acetic acid. Mobile phase solvent B consisted of a volumetric preparation of acetonitrile and 2-propanol in a 1:1 mixture. Separation was achieved using gradient elution: starting conditions were 90%A changing linearly to 35%B over the next 9.25min at 0.6ml/min, to 85%B within 2.25min at 0.6 ml/min, and then to 100%B within 0.3min at 0.8ml/min, at which it was kept for <1 min. Afterwards the solvent composition returned to starting conditions, followed by re-equilibration for 2.5min with increasing flow rate prior to the next injection.

Mass spectrometry was performed using electrospray in negative ESI ionization modes. The capillary voltage was 1.5kV, sampling and extraction cone voltages were 60V and 4V respectively, desolvation temperature was 600°C, and source temperature was 120°C. The cone gas flow rate was 150L/h, and desolvation gas flow rate was 1000L/h. The MS was operated in sensitivity mode with a scan time of 0.1s. For mass accuracy, a LockSpray interface was used with a 5ng/L leucine enkephalin (555.2645 amu) solution (50/50 ACN/H2O with 0.1% v/v formic acid) at 15µL/min was used as the lock mass. Data were collected in centroid mode with a scan range of 50−1200m/z, with lockmass scans collected every 30s and averaged over 4 scans to perform mass correction. The injection volume was 5µL and the auto-sampler was set at 4°C.

Tissue mRNA expression

Total RNA from duodenum, distal ileum and livers of mice was extracted using Qiazol lysis reagent or RLT buffer (both Qiagen, UK), and total RNA from human terminal ileal explants and gavaged murine terminal ileum was extracted using the RNeasy Mini Kit (Qiagen, UK) following bead beating with Qiagen Tissuelyser II, as per manufacturer's instructions. Reverse transcription was performed with the High-Capacity cDNA Reverse Transcription Kit, Thermo Fisher Scientific, UK. Real-time quantitative PCR was performed on Viia7 system (Thermo Fisher Scientific, UK), in a 384-well assay format using SYBR Green Mastermix (Sigma-Aldrich, UK). Primer sequences used are listed in Supplementary Table 1.

Tissue protein expression

Distal ileal ASBT protein levels were measured using western blotting. Protein was extracted by bead beating in phosphate-buffered saline and Halt Protease and Phosphatase Inhibitor Cocktail (Thermo Fisher Scientific, MA, USA), and quantified using Pierce BCA Protein Assay Kit (Thermo Fisher Scientific, MA, USA). 15µg protein per sample was separated by gel electrophoresis on NuPAGE 4-12% bis-tris protein gels (Thermo Fisher Scientific, MA, USA). Following transfer to membranes, protein was incubated with SLC10A2 antibody ab203205 (Abcam, Cambridge UK) and βactin

antibody 8H10D10 (Cell Signaling Technology, MA, USA) primary antibodies; secondary antibodies (anti-rabbit / anti-mouse respectively) were conjugated with horse radish peroxidase and developed using Pierce ECL western blotting substrate (Thermo Fisher Scientific, MA, USA). Protein concentrations were normalised to βactin, and compared with Mann-Whitney tests in GraphPad Prism.

Supplementary References

1. **Zhang JH**, **Nolan JD**, Kennie SL, Johnston IM, Dew T, Dixon PH, et al. Potent stimulation of fibroblast growth factor 19 expression in the human ileum by bile acids. Am J Physiol Liver Physiol 2013;304:G940–G948.
2. Bolger AM, Lohse M, Usadel B. Trimmomatic: a flexible trimmer for Illumina sequence data. Bioinformatics 2014;30:2114-2120.
3. Langmead B, Salzberg SL. Fast gapped-read alignment with Bowtie 2. Nat Methods 2010;9:357–359.
4. Edgar RC. Search and clustering orders of magnitude faster than BLAST. Bioinformatics 2010;26:2460–2461.
5. Lee JH, Yi H, Chun J. rRNASelector: A computer program for selecting ribosomal RNA encoding sequences from metagenomic and metatranscriptomic shotgun libraries. J Microbiol 2011;49:689–691.
6. **Caporaso JG**, **Kuczynski J**, **Stombaugh J**, Bittinger K, Bushman FD, Costello EK, et al. QIIME allows analysis of high-throughput community sequencing data. Nat Methods 2010:7;335–336.
7. DeSantis TZ, Hugenholtz P, Larsen N, Rojas M, Brodie EL, Keller K, et al. Greengenes, a Chimera-Checked 16S rRNA Gene Database and Workbench Compatible with ARB. Appl Environ Microbiol 2006;72:5069–5072.
8. Rho M, Tang H, Ye Y. FragGeneScan: predicting genes in short and error-prone reads. Nucleic Acids Res 2010;38:e191.
9. Jones P, Binns D, Chang HY, Fraser M, Li W, McAnulla C, et al. InterProScan 5: genome-scale protein function classification. Bioinformatics 2014;30:1236–1240.
10. Parks DH, Beiko RG. Identifying biologically relevant differences between metagenomic communities. Bioinformatics 2010;26:715–721.
11. Want EJ, Masson P, Michopoulos F, Wilson ID, Theodoridis G, Plumb RS, et al. Global metabolic profiling of animal and human tissues via UPLC-MS. Nat Protoc 2013;8:17-32.
12. Vorkas PA, Isaac G, Anwar MA, Davies AH, Want EJ, Nicholson JK, et al. Untargeted UPLC-MS profiling pipeline to expand tissue metabolome coverage: application to cardiovascular disease. Anal Chem 2015;87:4184-4193.
13. Spagou K, Wilson ID, Masson P, Theodoridis G, Raikos N, Coen M, et al. HILIC-UPLC-MS for exploratory urinary metabolic profiling in toxicological studies. Anal Chem 2011;83:382-390.
14. Sarafian MH, Lewis MR, Pechlivanis A, Ralphs S, McPhail MJW, Patel VC, et al. Bile acid profiling and quantification in biofluids using ultra-performance liquid chromatography tandem mass spectrometry. Anal Chem 2015;87:9662-9670.

Author names in bold designate shared co-first authorship

Supplementary Tables

Supplementary Table 1. Primer sequences used for qRT-PCR.

Gene	Abbreviation	Forward Primer Sequence (5' to 3')	Reverse Primer Sequence (5' to 3')
Murine			
Apical sodium dependent bile acid transporter	*Asbt*	TCCTGGCTAGACTAGCTGGTCAA	CTGAGTGTTCTGCATTCCAGTTTC
Bile salt export pump	*Bsep*	AAGCTACATCTGCCTTAGACAC	CAATACAGGTCCGACCCTCTCT
Cyclophilin b	*Cyclob*	TGGAGAGCACCAAGACAGACA	TGCCGGAGTCGACAATGAT
Cytochrome p450 7a1	*Cyp7a1*	AGCAACTAAACAACCTGCCAGTACTA	GTCCGGATATTCAAGGATGCA
Cytochrome p450 8b1	*Cyp8b1*	TAGCCCTCTTTCCTCCACTCAT	GAACCGATCGAACCTAAATTCCT
Fibroblast growth factor 15	*Fgf15*	GAGGACCAAAACGAACGAAATT	ACGTCCTTGATGGCAATCG
Farnesoid X receptor	*Fxr*	TCCGGACATTCAACCATCAC	TCACTGCACATCCCAGATCTC
Ileal bile acid-binding protein	*Ibabp*	TGAGAGTGAGAAGAATTACGATGAG TTC	TTACGTCCCCTTTCAATCACG
Organic solute transporter alpha	*Ostα*	CTGAGCATAGTGGGCCTGTTC	AGCTGCGCTCTTCTCAGAAATT
Organic solute transporter beta	*Ostβ*	TGACAAGCATGTTCCTCCTGAG	TTCTTTGTCTTGTGGCTGCTCC
Short heterodimer partner	*Shp*	CGATCCTCTTCAACCCAGATG	AGGGCTCCAAGACTTCACACA
Taurine transporter	*Taut*	GCACACGGCCTGAAGATGA	ATTTTTGTAGCAGAGGTACGGG
Human			
Fibroblast growth factor 19	*FGF19*	CGGTACCTCTGCATGGGC	CCATCTGGGCGGATCTCC
Farnesoid X receptor	*FXR*	AGGATTTCAGACTTTGGACCATGA	TGCCCAGACGGAAGTTTCTTATT
Intestinal bile acid binding protein	*IBABP*	TCAGAGATCGTGGGTGACAA	TCACGCGCTCATAGGTCA
Mitochondrial ribosomal protein L19	*L19*	CCAACTCCCGTCAGCAGATC	CAAGGTGTTTTTCCGGCATC
Organic solute transporter alpha	*OSTα*	AGATTGCTTGTTCGCCTCC	ATTCGTGTCAGCACAGTCATT
Organic solute transporter beta	*OSTβ*	GTGGAAGATGCATATCCCT	TTCTTCCCAGCAGGACCA
Short heterodimer partner	*SHP*	ATCCTCTTCAACCCCGATGTG	AAGGAAGGCCAGCGATGTCAA

Bile acids		Non-pregnant chow vs pregnant normal chow		Non-pregnant chow vs non-pregnant cholic acid		Non-pregnant cholic acid vs pregnant cholic acid		Pregnant chow vs pregnant cholic acid	
Species	Abbreviation	p-value	Fold change	p-value	Fold change	p-value	Fold change	p-value	Fold change
Cholic acid	CA	NS	-2.97	NS	5.87	NS	-1.84	NS	9.48
Chenodeoxycholic acid	CDCA	NS	-1.02	NS	-3.04	NS	-1.33	**1.08E-02**	-3.95
αMuricholic acid	α-MCA	NS	-1.53	**4.88E-04**	-12.26	NS	-1.36	**1.58E-02**	-10.87
βMuricholic acid	β-MCA	NS	-1.52	**1.10E-03**	-35.97	NS	-1.52	**2.39E-02**	-36.04
ωMuricholic acid	ω-MCA	NS	1.26	**3.05E-04**	-12.36	NS	-1.35	**1.44E-02**	-21.02
Deoxycholic acid	DCA	NS	1.27	**2.43E-03**	4.16	NS	-1.53	NS	-1.53
Hyocholic acid	HCA	NS	-1.32	NS	3.73	NS	1.41	**1.82E-02**	6.91
Hyodeoxycholic acid	HDCA	NS	1.16	**1.38E-02**	-5.57	NS	1.43	**1.83E-02**	-4.53
Lithocholic acid	LCA	NS	-1.20	**1.90E-04**	-8.59	NS	-1.01	**2.44E-02**	-7.25
Murocholic acid	MDCA	NS	-1.75	**5.36E-03**	-15.88	NS	1.01	**1.67E-02**	-8.99
Ursodeoxycholic acid	UDCA	NS	1.28	**2.02E-03**	-7.24	NS	2.35	**1.13E-02**	-3.92
5β-Cholanoic acid 3β, 12α-diol	iso-DCA	NS	1.24	**1.39E-02**	3.06	NS	1.19	**1.78E-02**	2.94
Taurocholic acid	TCA	NS	-2.39	NS	3.13	NS	-1.94	NS	3.87
Taurodeoxycholic acid	TDCA	**1.28E-02**	-3.00	NS	3.78	NS	-1.22	NS	9.28
Taurohyodeoxycholic acid	THDCA	**3.31E-02**	-3.97	**8.98E-03**	-9.40	NS	2.39	NS	1.01
Tauromuricholic acids	TMCAs	NS	-7.96	**2.37E-02**	-71.67	NS	2.51	NS	-3.59
Tauroursodeoxycholic acid	TUDCA	**3.46E-02**	-6.79	**1.15E-02**	-81.10	NS	5.23	NS	-2.29
Glycocholic acid	GCA	NS	-2.36	NS	18.51	NS	2.18	NS	95.36
Glycodeoxycholic acid	GDCA	NS	-1.23	NS	11.63	NS	-1.31	NS	10.88
Taurocholic acid sulfate	TCA-S	**3.58E-03**	-77.96	**2.43E-03**	542.69	NS	1.02	NS	-6.83
Taurocholic acid sulfate	TCA-S	**4.87E-04**	-46.27	**3.45E-04**	-452.99	NS	-1.21	NS	-11.87
Cholic acid sulfate	CA-S	NS	1.80	**5.05E-03**	4.99	NS	1.45	**1.56E-02**	4.01

Supplementary Table 2. Bile acid species in cecal content as determined by UPLC-MS/MS. Results show significance by Student’s t test with Benjamini-Hochberg correction, where p<0.05 and fold change of bile acid species when compared between pregnancy and diet groups. NS – not significant

Taxonomy	Non-pregnant chow %	Pregnant chow %	Non-pregnant cholic acid %	Pregnant cholic acid %
Unassigned; Other; Other; Other; Other; Other	3.7	1.9	3.2	1.9
k_Bacteria; p_Bacteroidetes; c_Bacteroidia; o_Bacteroidales; Other; Other	1.1	1.5	1.2	1.2
k_Bacteria; p_Bacteroidetes; c_Bacteroidia; o_Bacteroidales; f_; g_	0	2.7	0.6	0.9
k_Bacteria; p_Bacteroidetes; c_Bacteroidia; o_Bacteroidales; f_Bacteroidaceae; g_Bacteroides	0	2.5	16.7	14.7
k_Bacteria; p_Bacteroidetes; c_Bacteroidia; o_Bacteroidales; f_Porphyromonadaceae; g_Parabacteroides	5.3	0	1.4	0.8
k_Bacteria; p_Bacteroidetes; c_Bacteroidia; o_Bacteroidales; f_Prevotellaceae; g_Prevotella	0	0.9	5.8	3.1
k_Bacteria; p_Bacteroidetes; c_Bacteroidia; o_Bacteroidales; f_Rikenellaceae; g_	0	7.5	7.2	11.7
k_Bacteria; p_Bacteroidetes; c_Bacteroidia; o_Bacteroidales; f_S24-7; g_	13.0	27.0	31.8	25.3
k_Bacteria; p_Bacteroidetes; c_Bacteroidia; o_Bacteroidales; f_Odoribacteraceae; g_Odoribacter	0	2.0	0.3	0.7
k_Bacteria; p_Bacteroidetes; c_Bacteroidia; o_Bacteroidales; f_Paraprevotellaceae; g_Prevotella	0	0.2	2.1	0.1
k_Bacteria; p_Cyanobacteria; c_Chloroplast; o_Streptophyta; f_; g_	1.9	0	0.8	0
k_Bacteria; p_Deferribacteres; c_Deferribacteres; o_Deferribacterales; f_Deferribacteraceae; g_Mucispirillum	4.3	2.9	3.0	1.8
k_Bacteria; p_Firmicutes; c_Clostridia; o_Clostridiales; f_; g_	39.2	26.4	8.3	16.9
k_Bacteria; p_Firmicutes; c_Clostridia; o_Clostridiales; f_Lachnospiraceae; Other	1.5	2.2	0.8	1.3
k_Bacteria; p_Firmicutes; c_Clostridia; o_Clostridiales; f_Lachnospiraceae; g_	7.7	4.8	2.7	2.3
k_Bacteria; p_Firmicutes; c_Clostridia; o_Clostridiales; f_Ruminococcaceae; Other	1.4	1.2	1.1	1.4
k_Bacteria; p_Firmicutes; c_Clostridia; o_Clostridiales; f_Ruminococcaceae; g_	2.5	2.8	1.7	3.2
k_Bacteria; p_Firmicutes; c_Clostridia; o_Clostridiales; f_Ruminococcaceae; g_Oscillospira	3.4	4.1	2.0	2.9
k_Bacteria; p_Proteobacteria; c_Alphaproteobacteria; o_RF32; f_; g_	0	0.1	1.2	0.6
k_Bacteria; p_Proteobacteria; c_Deltaproteobacteria; o_Desulfovibrionales; f_Desulfovibrionaceae; g_Bilophila	0	0.5	1.4	1.1
k_Bacteria; p_Tenericutes; c_Mollicutes; o_Anaeroplasmatales; f_Anaeroplasmataceae; g_Anaeroplasma	8.4	2.8	0	0
k_Bacteria; p_Verrucomicrobia; c_Verrucomicrobiae; o_Verrucomicrobiales; f_Verrucomicrobiaceae; g_Akkermansia	0	0	0.8	3.0

Supplementary Table 3. Bacterial taxonomy to genus level of microbiota of cecal content. Per cent of total 16S rRNA sequences derived from metagenomic analysis for each diet and pregnancy group displayed, where abundance was >1.00%. k – kingdom, p – phylum, c – class, o – order, f – family, g – genus.

Analysis	Components (predictive + orthogonal)	R2X (cum)	R2Y (cum)	Q2 (cum)	CV-ANOVA
**Bile acid profiling ESI-**	3+2	0.738	0.953	0.873	2.74E-17
**Lipid profiling ESI+**	3+2	0.651	0.924	0.790	2.12E-10
**Lipid profiling ESI-**	3+1	0.621	0.921	0.817	6.93E-16
**HILIC analysis of aqueous extracts ESI+**	3+1	0.646	0.930	0.764	1.51E-14
**HILIC analysis of aqueous extracts ESI-**	3+1	0.699	0.939	0.837	6.10E-20

Supplementary Table 4. Summary of model characteristics from O2PLS-DA multivariate statistical analyses of data obtained from all analyses and electrospray ionization polarity modes. CV-ANOVA, cross-validation ANOVA testing of residual values from cross-validation testing.

	Pathway	Non-pregnant chow vs pregnant chow	Non-pregnant chow vs non-pregnant cholic acid
Colonizers – metagenomics			
Amino acid metabolism	Lysine biosynthesis	Y	N
	Arginine and proline metabolism	Y	Y
	Cysteine and methionine metabolism	N	Y
Lipid metabolism	Sphingolipid metabolism	Y	N
	α-linoleic acid metabolism	Y	Y
	Fatty acid biosynthesis	N	Y
	Ether lipid metabolism	N	Y
Carbohydrate metabolism	Glycolysis / gluconeogenesis	Y	Y
	Citrate cycle (TCA cycle)	Y	Y
	Fructose and mannose metabolism	Y	Y
	Pentose phosphate pathway	Y	Y
Nucleotide metabolism	Pyrimidine metabolism	Y	Y
	Purine metabolism	N	Y
Glycan biosynthesis and metabolism	Other glycan degradation	Y	Y
	Glycosaminoglycan degradation	Y	Y
	Lipopolysaccharide biosynthesis	Y	Y
	Glycosylphosphatidylinositol-anchor biosynthesis (cell surface)	Y	Y
	Glycosphingolipid biosynthesis – lacto and neolacto series	Y	Y
	Glycosphingolipid biosynthesis – globo series	Y	Y
	Glycosphingolipid biosynthesis – ganglio series	Y	Y
	Peptidoglycan biosynthesis	N	Y
Metabolism of cofactors and vitamins	One carbon pool by folate	Y	Y
	Thiamine metabolism	Y	Y
	Riboflavin metabolism	Y	Y
	Vitamin B6 metabolism	Y	Y
	Folate biosynthesis	Y	Y

	Ubiquinone and other terpenoid-quinone biosynthesis	N	Y
	Pantothenate and CoA biosynthesis	N	Y
Energy metabolism	Carbon fixation in photosynthetic organisms	Y	Y
	Sulfur metabolism	N	Y
Xenobiotic biodegradation and metabolism	Drug metabolism – other enzymes	Y	Y
	Polycyclic aromatic hydrocarbon degradation	N	Y
	Naphthalene degradation	N	Y
Metabolism of other amino acids	β-alanine metabolism	N	Y
	Selenocompound metabolism	N	Y
	Glutathione metabolism	N	Y
Metabolism of terpenoids and polyketides	Limonene and pinene degradation	N	Y
Translation	Aminoacyl-tRNA biosynthesis	N	Y
Host – metagenomics			
Liver	Liver necrosis / cell death	Y	Y
	Glutathione depletion in liver	Y	Y
	Liver damage	Y	Y
	Liver steatosis	Y	N
	Liver fibrosis	Y	Y
	Hepatocellular carcinoma	Y	Y
	Liver hyperplasia / hyperproliferation	Y	Y
	Liver cholestasis	Y	Y
	Liver inflammation / hepatitis	Y	Y
	Liver cirrhosis	N	Y
	Biliary hyperplasia	Y	Y
Kidney	Renal fibrosis	Y	N
	Renal damage	Y	N
	Renal dilation	Y	N
	Renal hypertrophy	N	Y
	Renal dysfunction	Y	N
	Kidney failure	Y	Y
	Nephrosis	N	Y
	Glomerular injury	Y	Y
Heart	Cardiac damage	Y	Y
	Cardiac arrhythmia	Y	Y
	Cardiac arteriopathy	Y	N
	Bradycardia	Y	Y
Biochemistry	Increased levels of bilirubin	N	Y
	Increased levels of lactate dehydrogenase	Y	Y
	Increased levels of blood urea nitrogen	Y	N
	Increased levels of albumin	Y	N
	Increased levels of alkaline phosphatase	N	Y
	Increased levels of creatinine	Y	Y

Supplementary Table 5. Results of pathway analyses of metabolite and metagenomics changes in murine cecal content for mice fed chow and cholic acid supplemented diets, when not-pregnant and pregnant. Analyses were performed using Ingenuity Pathway Analysis (IPA) for host and an internal selection criterion for colonizers. Y=Yes, pathway differs between compared groups; N=No, no pathway difference between compared groups.

Supplementary Figures

Supplementary Figure 1. Workflows for data analyses

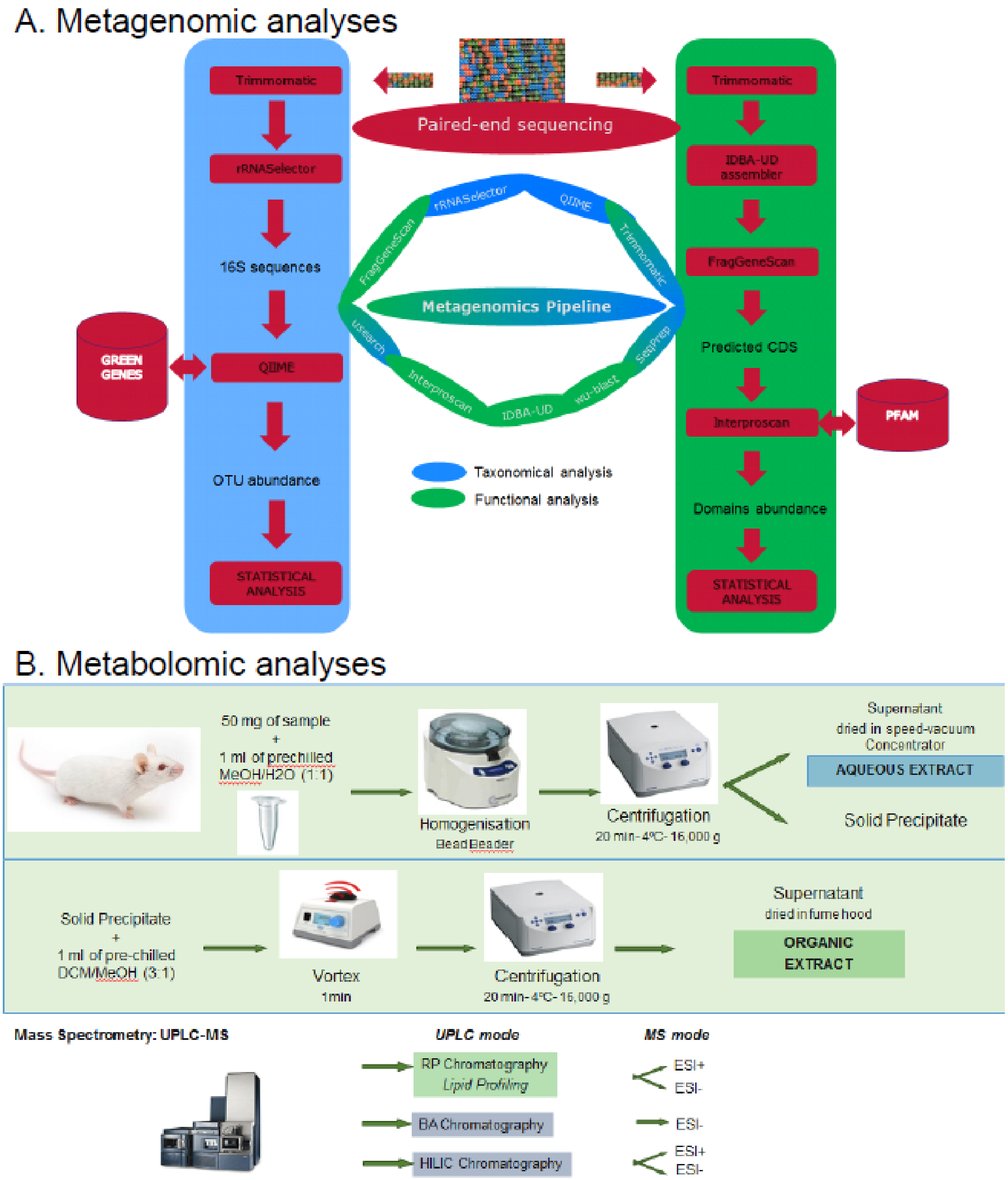

Supplementary Figure 2.

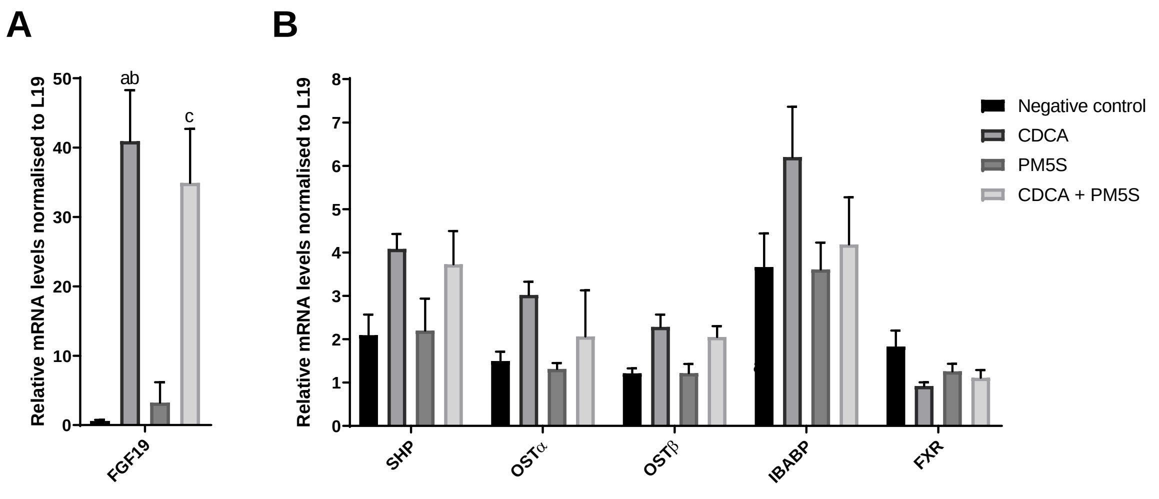

(A) FGF19 and **(B)** small heterodimer partner (SHP), organic solute transporter (OST)α, OSTβ, ileal bile acid-binding protein (IBABP) and farnesoid X receptor (FXR) mRNA expression relative to L19 in human terminal ileal explants cultured with chenodeoxycholic acid (CDCA) and/or epiallopregnanolone sulfate (PM5S). Bars show mean + SEM. Significance determined by $p<0.05$; a: CDCA compared with negative control, b: CDCA compared with PM5S, c: CDCA+PM5S compared with PM5S.

Supplementary Figure 3. Murine distal ileal mRNA expression following PM5S oral gavage

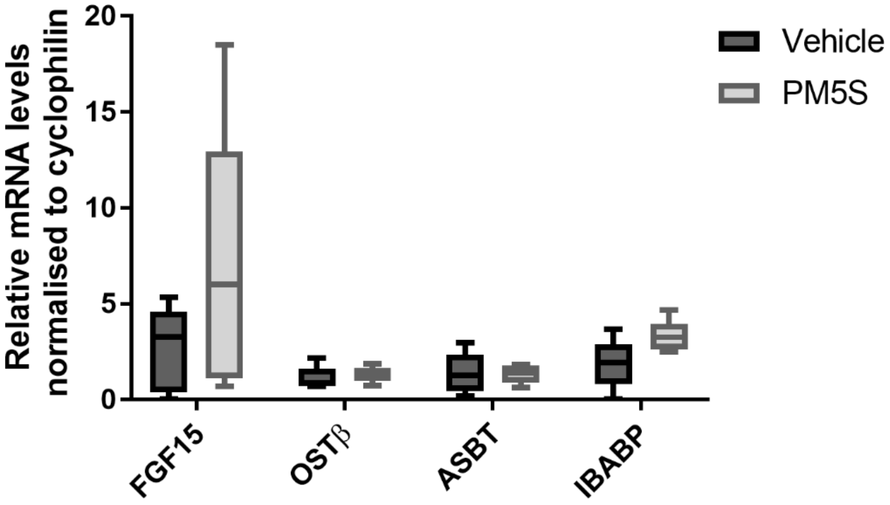

Expression levels of FGF15, organic solute transporter (OST)β, ASBT and ileal bile acid binding protein (IBABP) mRNA in murine distal ileum of mice following gavage with vehicle or epiallopregnanolone sulfate (PM5S), assessed by multiple measures of ANOVA with Tukey post hoc testing. Boxes show IQR with whiskers at 1.5 IQR, N=5.

Supplementary Figure 4. Murine hepatic mRNA expression before and during pregnancy

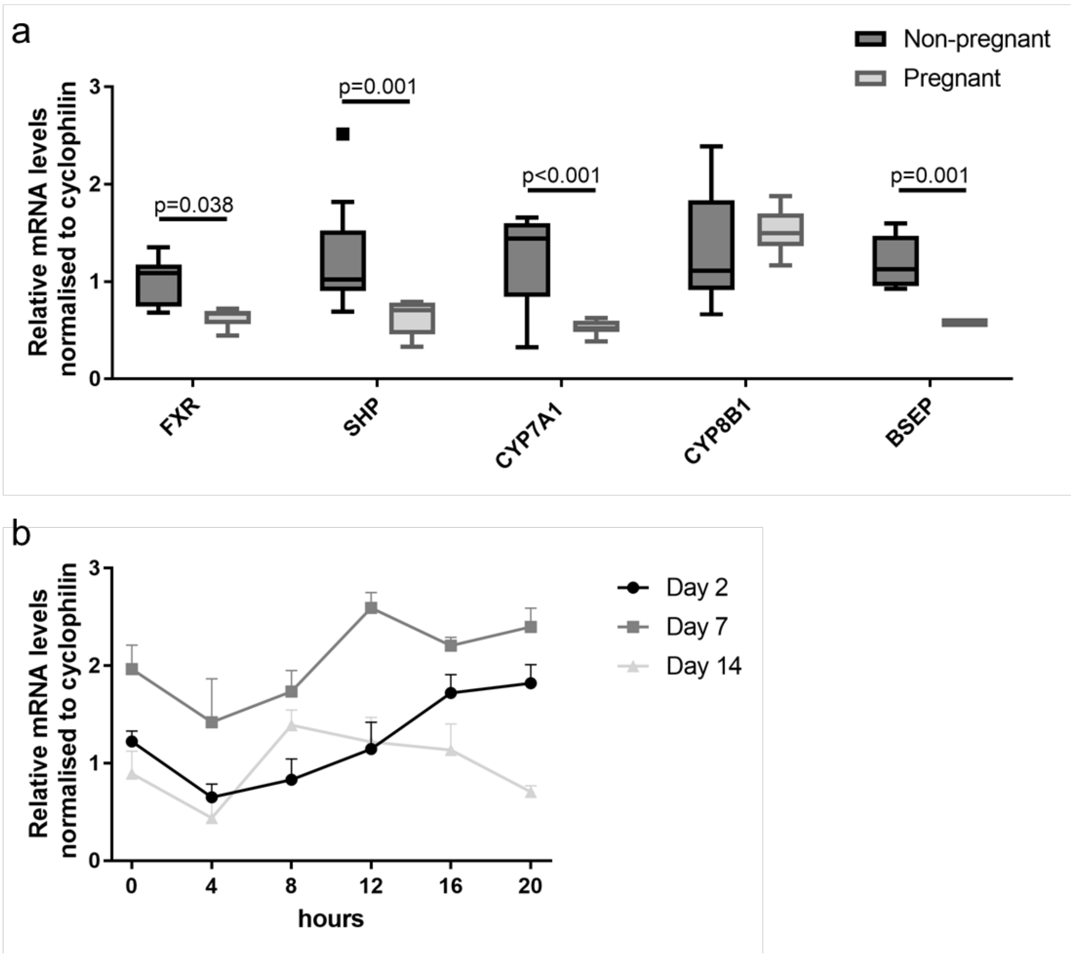

(A) Expression levels of hepatic FXR, SHP, CYP7A1, CYP8B1 and BSEP for non-pregnant and pregnant mice (gestational day18). Groups compared with 2-way ANOVA with Holm-Sidak's multiple comparison testing. Boxes show IQR with whiskers at 1.5 IQR, N=6-10.

(B) Expression levels of hepatic CYP7A1 mRNA over 24 hours for mice at gestational days 2, 7 and 14. Line graphs show mean + SEM, N=6-7 for each time-point.

Supplementary Figure 5. Murine hepatic and gall bladder bile acid levels.

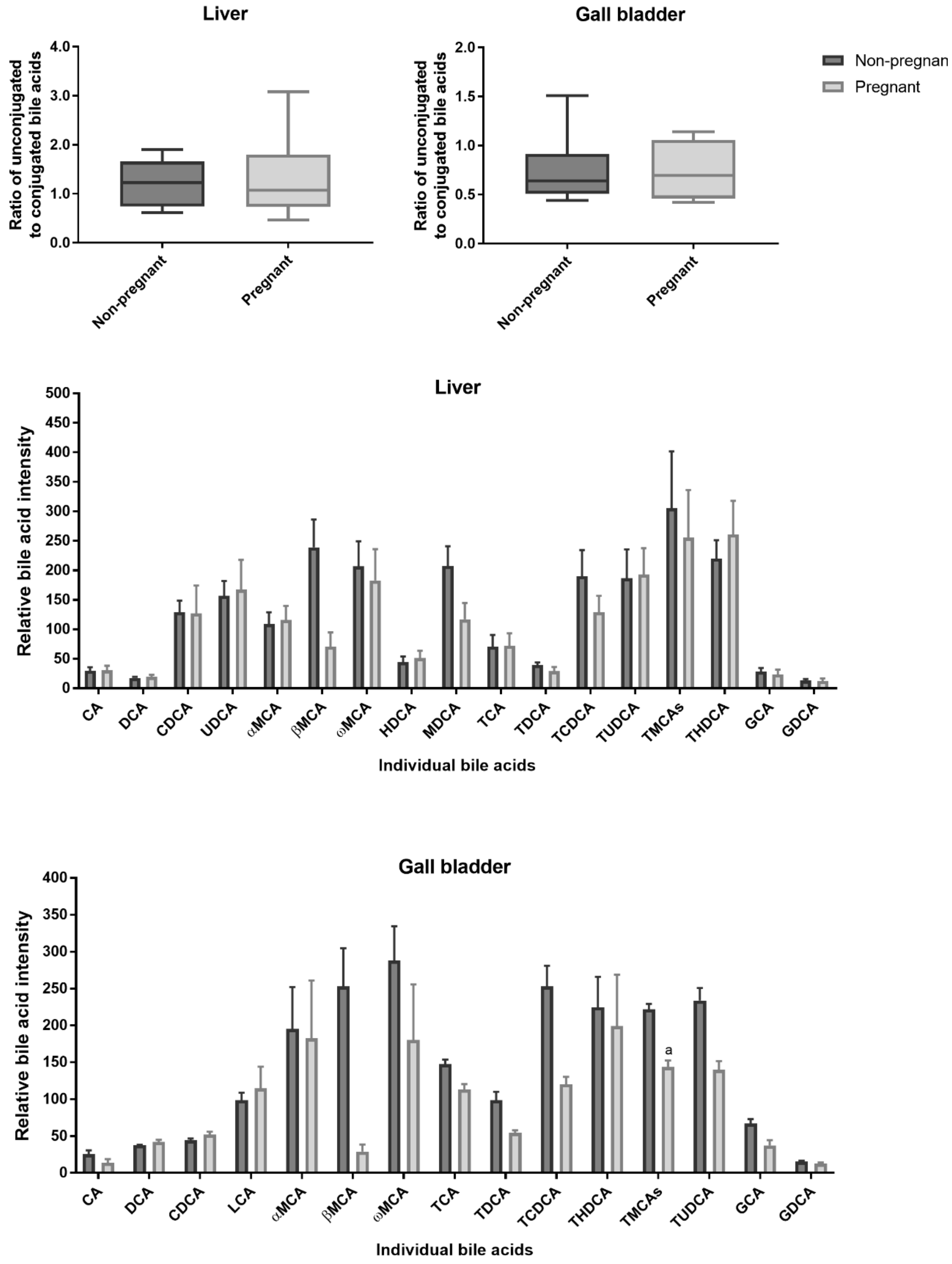

Relative bile acid conjugation (unconjugated:conjugated) in murine liver and gall bladders from non-pregnant and pregnant mice. Conjugation ratios compared with Mann-Whitney tests, and individual bile acids compared with multiple t tests, with Holm-Sidak method to correct for multiple comparisons, a indicates significant ($p<0.05$) between groups. Boxes show IQR with whiskers at 1.5IQR, bars show mean + SEM, N=4-10.

Supplementary Figure 6. Toxicological functions differ in murine cecal content from mice fed normal and cholic acid-supplemented diets, according to gestation

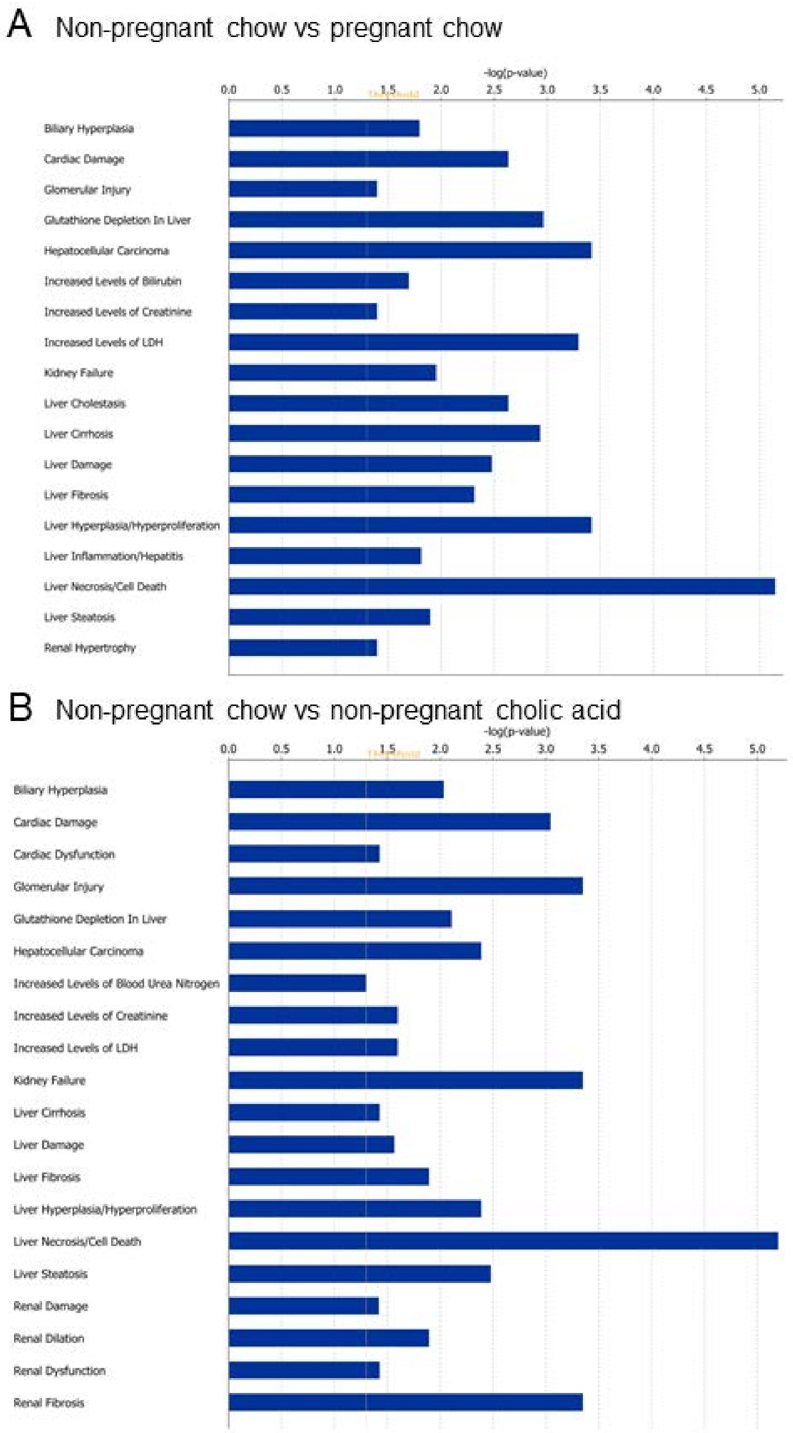

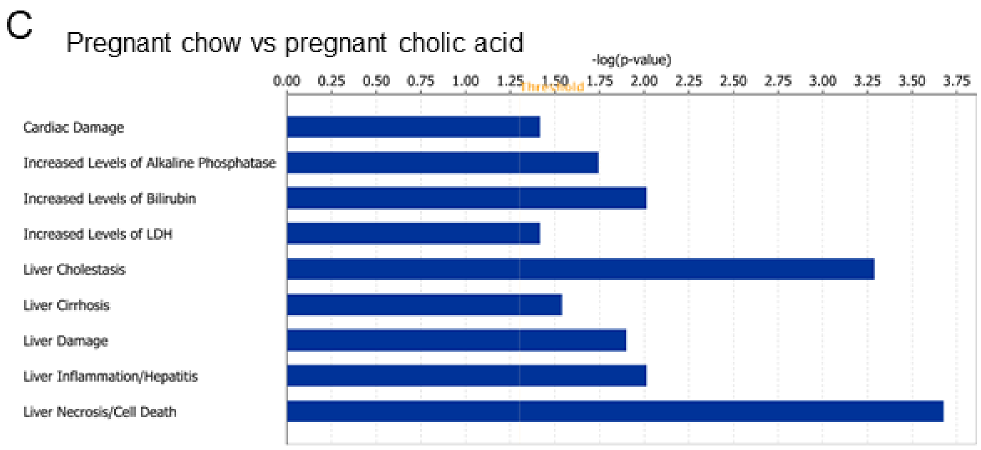

Toxicological functions selected by Ingenuity Pathway Analysis.

(A) Non-pregnant chow vs pregnant chow diet

(B) Non-pregnant chow vs non-pregnant cholic acid-supplemented diet

(C) Pregnant chow vs pregnant cholic acid-supplemented diets

Supplementary Figure 7. Diseases and biofunctions differ in murine cecal content from mice fed normal and cholic acid-supplemented diets, according to gestation

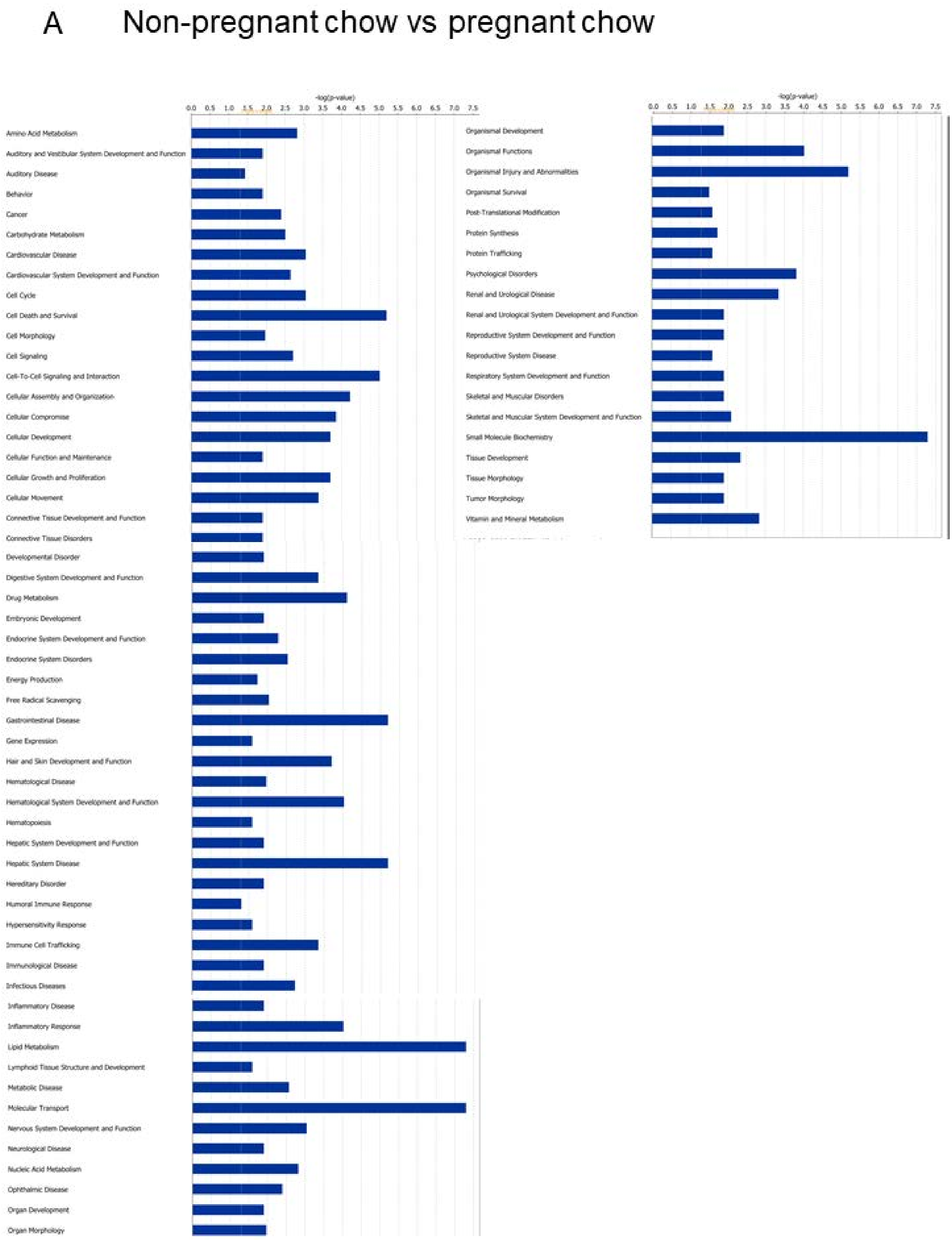

B Non-pregnant chow vs non-pregnant cholic acid

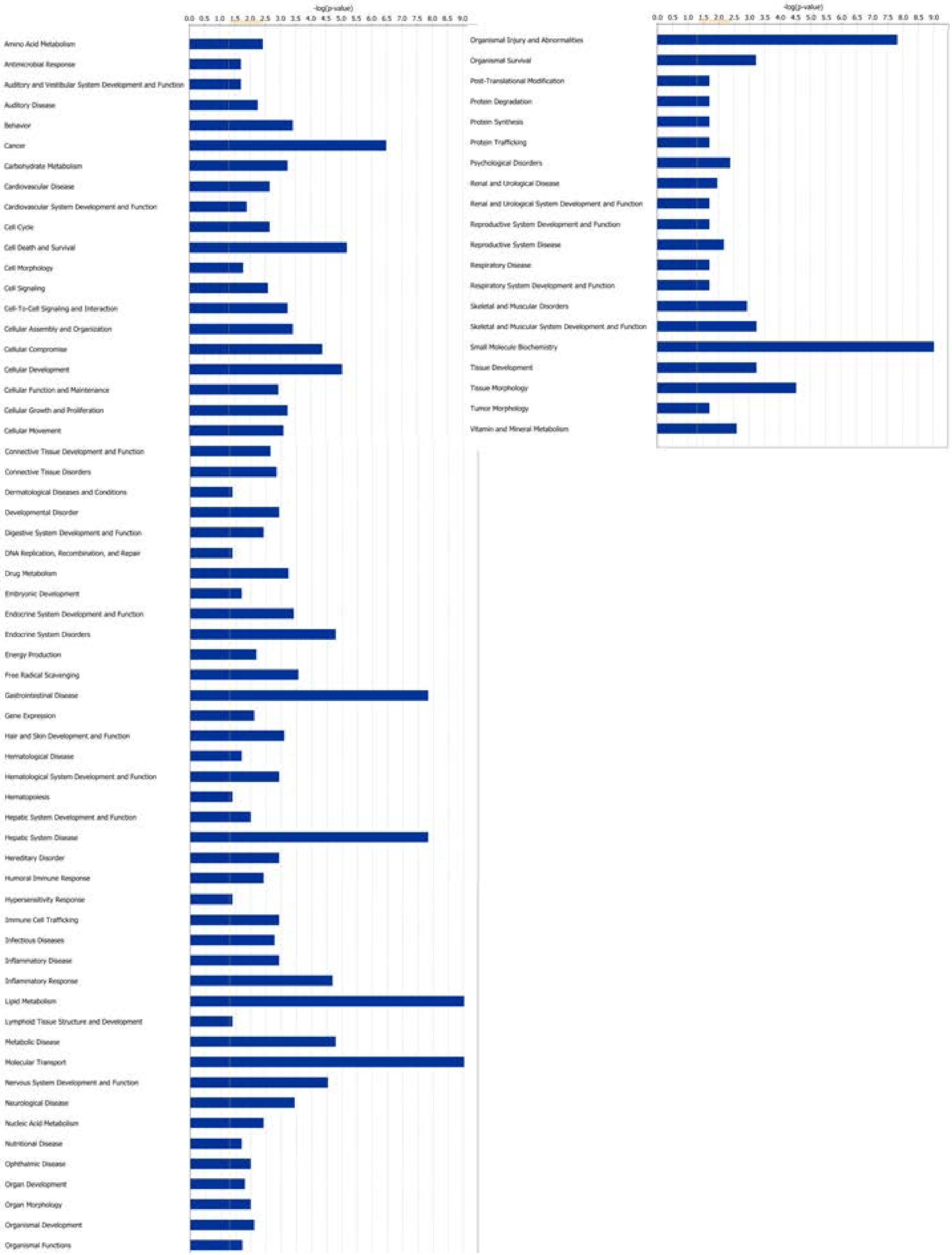

C Pregnant chow vs Pregnant cholic acid

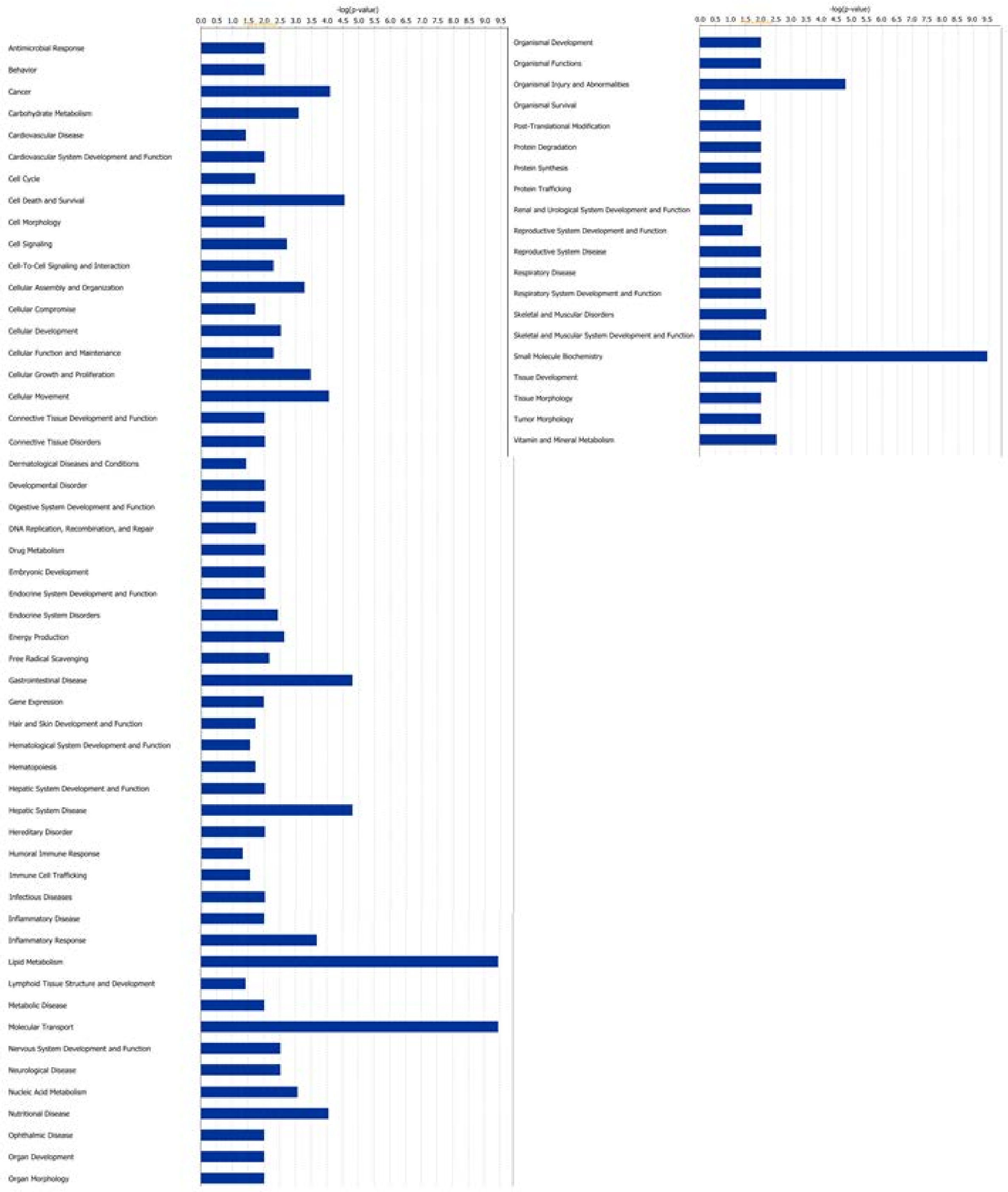

Diseases and biofunctions selected by Ingenuity Pathway Analysis.

(A) Non-pregnant chow vs pregnant chow diet

(B) Non-pregnant chow vs non-pregnant cholic acid-supplemented diet

(C) Pregnant chow vs pregnant cholic acid-supplemented diet

Supplementary Figure 8. Metabolic pathways differ in murine cecal content from mice fed normal and cholic acid-supplemented diets, according to gestation

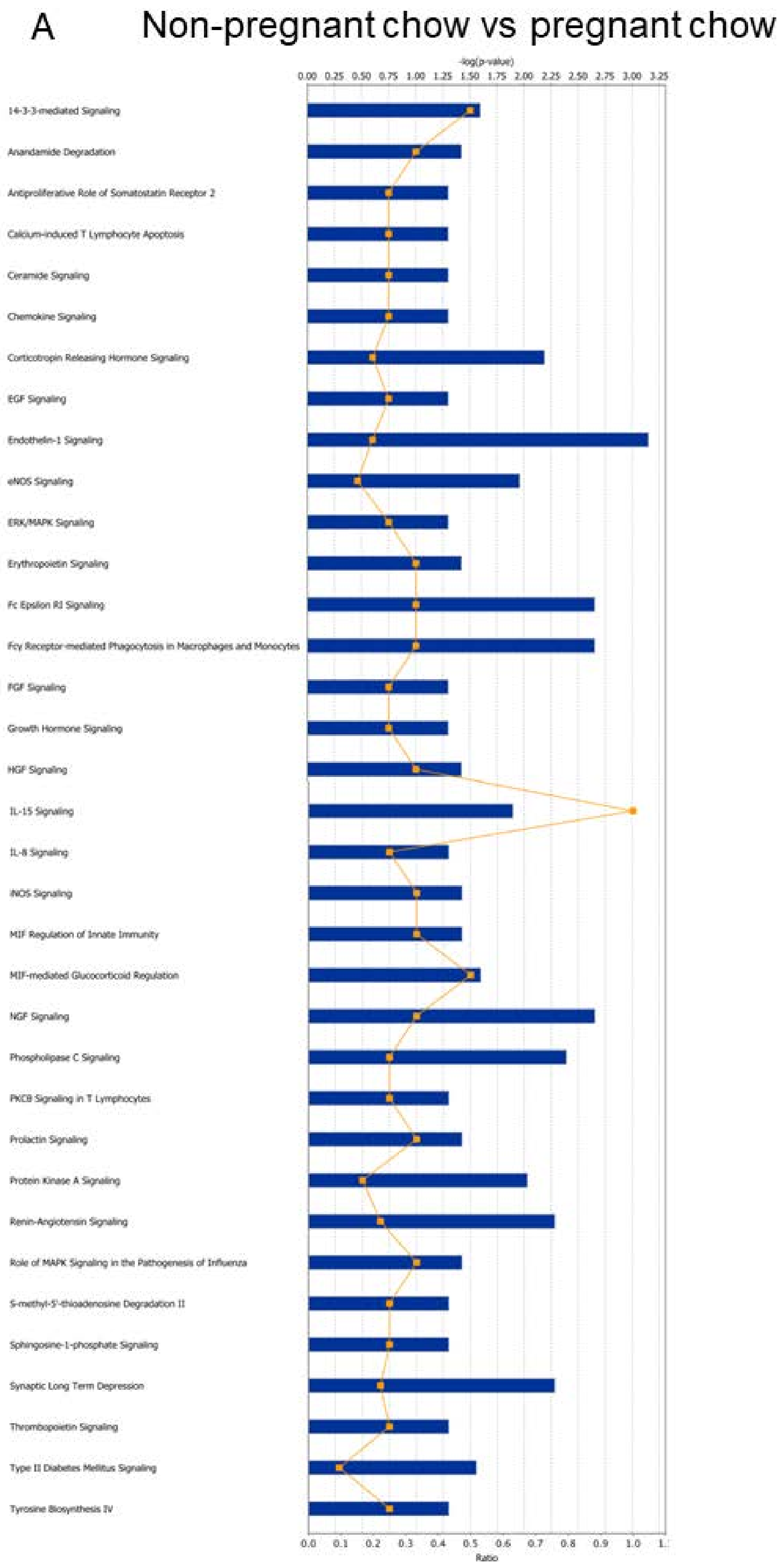

B Non-pregnant chow vs non-pregnant cholic acid

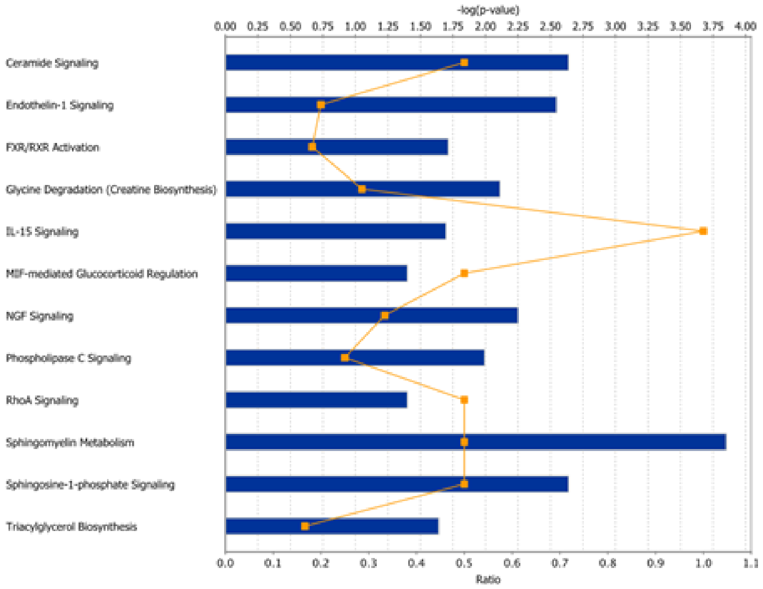

C Pregnant chow vs Pregnant cholic acid

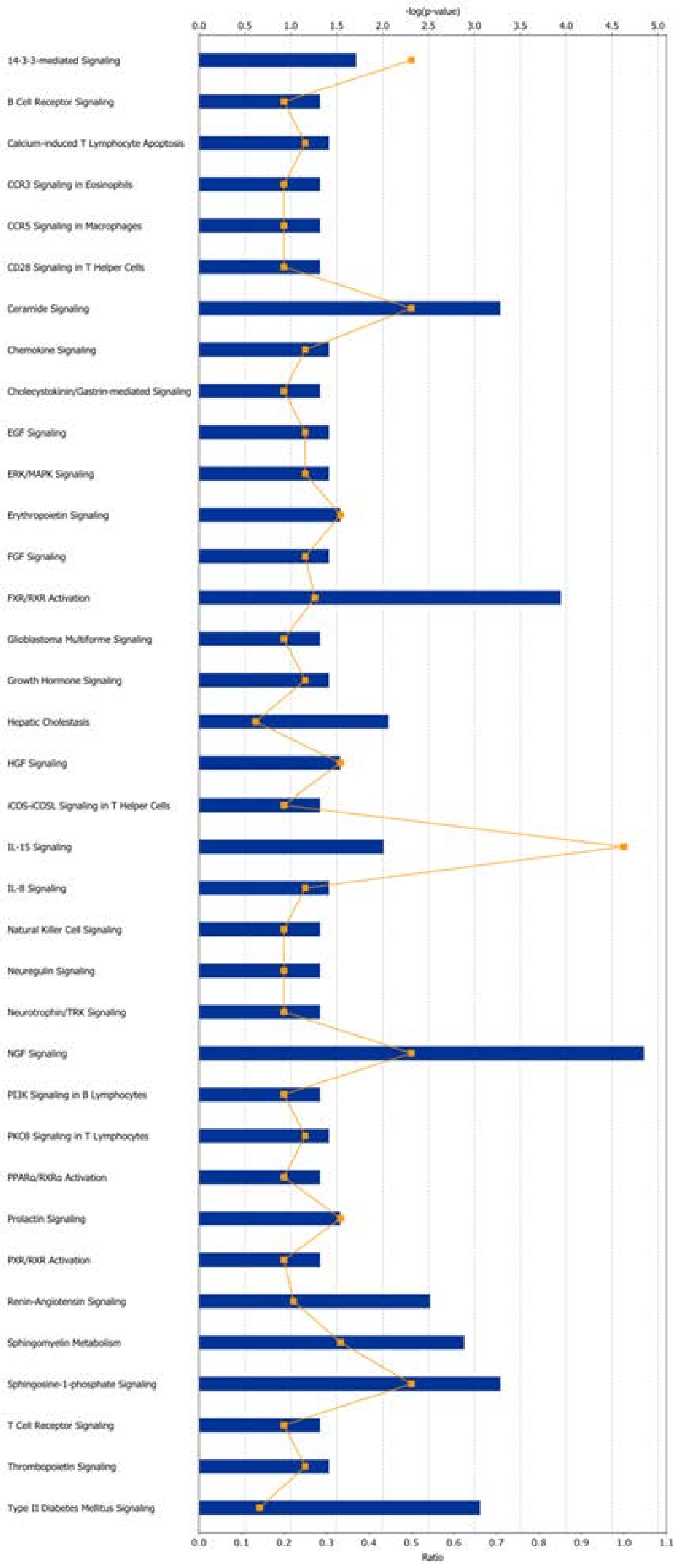

Canonical pathways selected by Ingenuity Pathway Analysis.

(A) Non-pregnant chow vs pregnant chow diet

(B) Non-pregnant chow vs non-pregnant cholic acid-supplemented diet

(C) Pregnant chow vs pregnant cholic acid-supplemented diet

Supplementary Figure 9. Glycine cleavage system enzymes selected in KEGG and protein domain pathway analyses as being elevated in pregnancy and cholic acid feeding

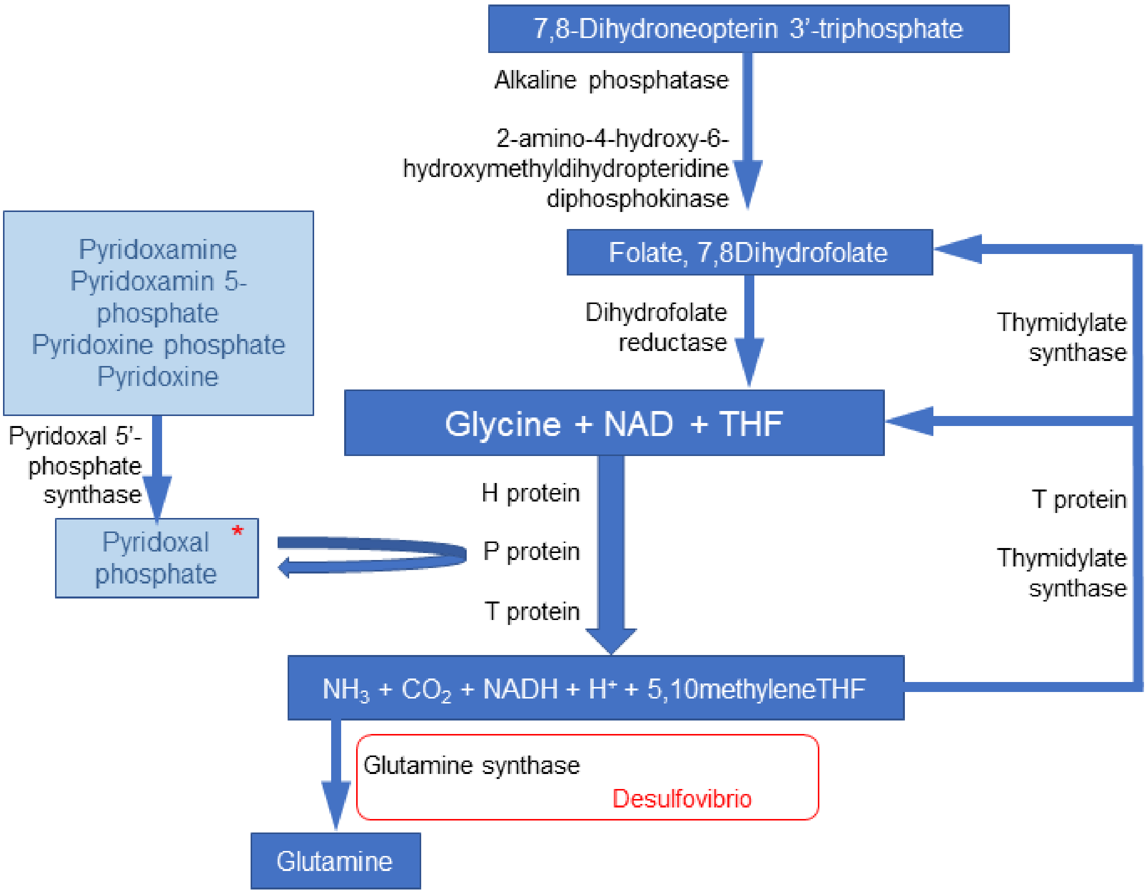

Enzymes represented in black font, cofactor for P protein in pale blue. Pathways simplified for diagrammatic representation, with only the enzymes differing between pregnancy and diet groups represented on the figure. *pyridoxal phosphate is marked as it is also a cofactor in carnitine biosynthesis, a side-product of which is also glycine. NAD: nicotinamide adenine dinucleotide, THF: tetrahydrofolate
